# Supplementary material for: A flexible loop in the paxillin LIM3 domain mediates its direct binding to integrin β subunits
Source: PLoS Biol. 2024 Sep 4;22(9):e3002757. doi: 10.1371/journal.pbio.3002757 (PMC11374337; doi:10.1371/journal.pbio.3002757)
Supplement: S1 Fig — (A) Schematic overview of the OPTIC workflow. Opa-expressing Ngo are used to cluster CEACAM3-integrin β cytoplasmic tail fusion proteins potentially resulting in the recruitment of an intracellular protein of interest (POI). (B) 293T cells were transiently cotransfected with a CEACAM3-ITGB1 (ITGB1) or CEACAM3-ITGB3 (ITGB3) fusion construct together with GFP-labelled LIM-domain-containing proteins. WCLs of the transfected 293T cells were probed by western blotting with a monoclonal antibody against GFP to detect the expression of GFP-LIM proteins (upper panel). Coomassie staining (lower panel) was used to verify equal loading of the membrane. (C) 293T cells transfected with CEACAM3-ITGB1 (ITGB1) and the indicated GFP-fusion proteins were seeded on poly-L-lysine. Cells were infected for 1 h with Pacific Blue−labelled Neisseria gonorrhoeae (Ngo; blue), fixed, and stained for ITGB1 (red). Recruitment of GFP-LIM proteins to clustered ITGB1 tail is indicated by white arrowheads. Bars represent 2 μm. (D) Quantification of (C). Each data point reflects the recruitment ratio R in a CEACAM3-ITGB1-expressing cell with associated bacteria. Horizontal lines indicate mean values and 95% confidence intervals (whiskers) of n = 60 cells from 3 independent experiments. Statistical significance was calculated using one-way ANOVA, followed by Bonferroni multiple comparison test (*** p < 0.001, ns = not significant). The data underlying this panel can be found in S1 Data. (E) Quantification and statistical evaluation as in (D) of GFP-fusion protein recruitment to CEACAM3-ITGB3 (ITGB3) (see main Fig 1A). The data underlying this panel can be found in S1 Data. (PDF) [file pbio.3002757.s001.pdf]

Baade et al., Suppl. Fig. S1

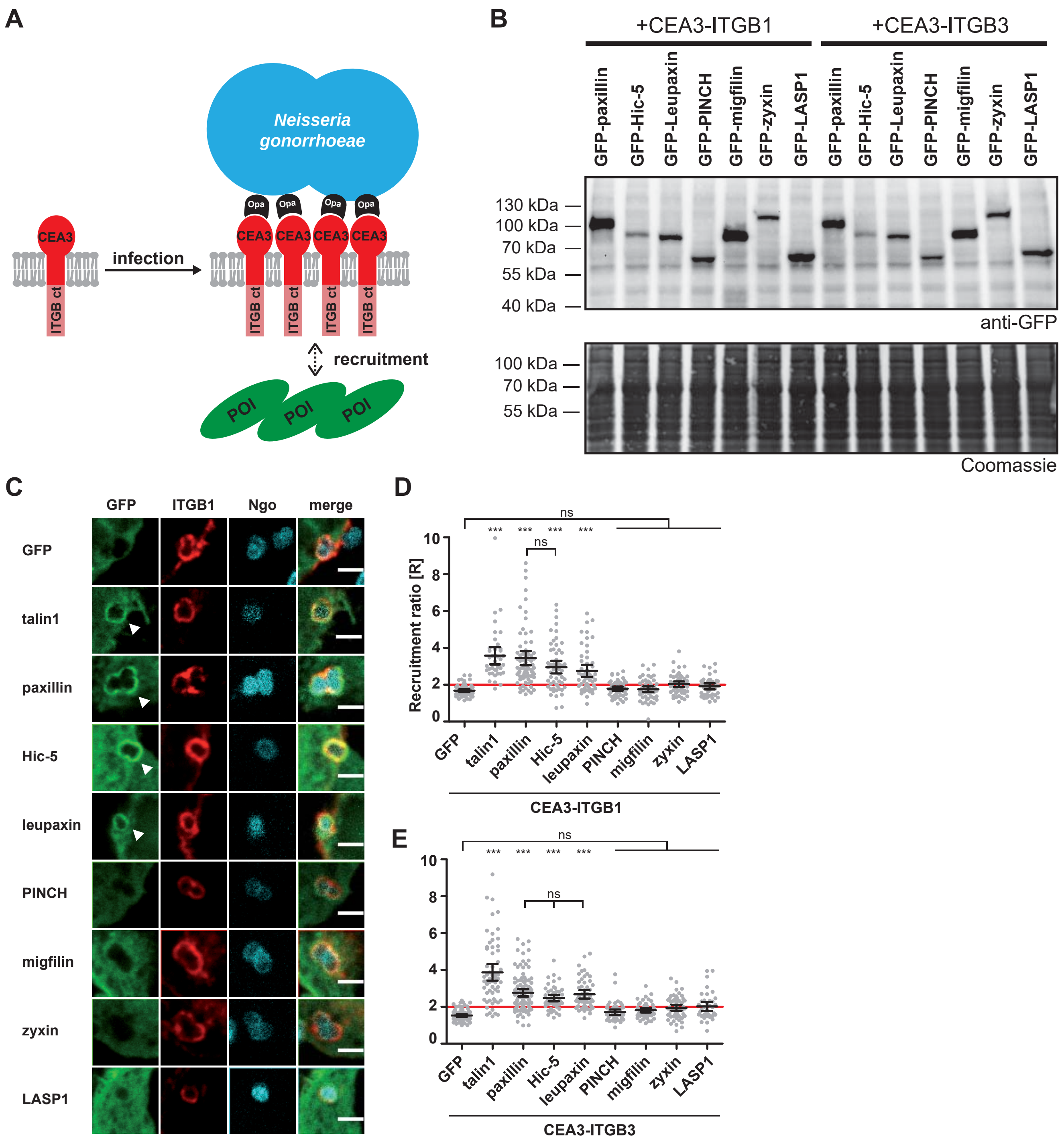

Supplementary Figure S1: Paxillin and closely related LIM-domain proteins localize to clustered integrin-β1 or -β3 ct.

(A) Schematic overview of the OPTIC workflow. Opa-expressing Ngo are used to cluster CEACAM3-integrin β cytoplasmic tail fusion proteins potentially resulting in the recruitment of an intracellular protein of interest (POI). (B) 293T cells were transiently co-transfected with a CEACAM3-ITGB1 (ITGB1) or CEACAM3-ITGB3 (ITGB3) fusion construct together with GFP labelled LIM-domain-containing proteins. Whole cell lysates of the transfected 293T cells were probed by Western Blotting with a monoclonal antibody against GFP to detect the expression of GFP-LIM proteins (upper panel). Coomassie staining (lower panel) was used to verify equal loading of the membrane. (C) 293T cells transfected with CEACAM3-ITGB1 (ITGB1) and the indicated GFP-fusion proteins were seeded on poly-L-lysine. Cells were infected for 1 h with Pacific Blue labelled *Neisseria gonorrhoeae* (Ngo; blue), fixed, and stained for ITGB1 (red). Recruitment of GFP-LIM proteins to clustered ITGB1 tail is indicated by white arrowheads. Bars represent 2 µm. (D) Quantification of (C). Each data point reflects the recruitment ratio R in a CEACAM3-ITGB1-expressing cell with associated bacteria. Horizontal lines indicate mean values and 95% confidence intervals (whiskers) of n = 60 cells from three independent experiments. Statistical significance was calculated using one-way ANOVA, followed by Bonferroni Multiple Comparison Test (*** $p<0.001$, ns = not significant). The data underlying this panel can be found in S1_Data.xlsx. (E) Quantification and statistical evaluation as in (D) of GFP-fusion protein recruitment to CEACAM3-ITGB3 (ITGB3) (see main Figure 1A). The data underlying this panel can be found in S1_Data.xlsx.
