# Supplementary material for: A flexible loop in the paxillin LIM3 domain mediates its direct binding to integrin β subunits
Source: PLoS Biol. 2024 Sep 4;22(9):e3002757. doi: 10.1371/journal.pbio.3002757 (PMC11374337; doi:10.1371/journal.pbio.3002757)
Supplement: S2 Fig — (A) Heteronuclear 15N{1H} NOE. The intensity ratio between spectra with and without 1H saturation is displayed vs. residue number. Values of 0.8 indicate rigid parts of the structure. Values smaller than 0.8 indicate increasing flexibility on the ps-to-ns timescale. The data underlying this panel can be found in S1 Data. On top of the heteronuclear NOE plot, the domain and secondary structure arrangement of the paxillin construct used in this study is depicted. α-Helices are shown in light blue. β-Sheets as magenta arrows. The secondary structure elements depicted here were identified with Pymol’s dss command and correspond to the cartoon representation of the 3D structural ensemble shown in Fig 2. The linker connecting the LIM2 with the LIM3 domain is shown in green. Regions harboring flexible loops are shown in blue and grey, respectively. (B) Sequence alignment of paxillin LIM3 domain across different species: Alignment was performed using the structural alignment tool from T-Coffee and coloured using the BoxShade tool at ExPASy. Identical residues are shaded in black; highly similar residues are shaded gray. In addition, an alignment is shown of paxillin’s LIM2 and LIM3 domains. The region harboring a flexible loop, which is crucial for binding of the LIM3 domain to β-integrin, is indicated by a blue line. (PDF) [file pbio.3002757.s002.pdf]

Baade et al., Suppl. Fig. S2

A

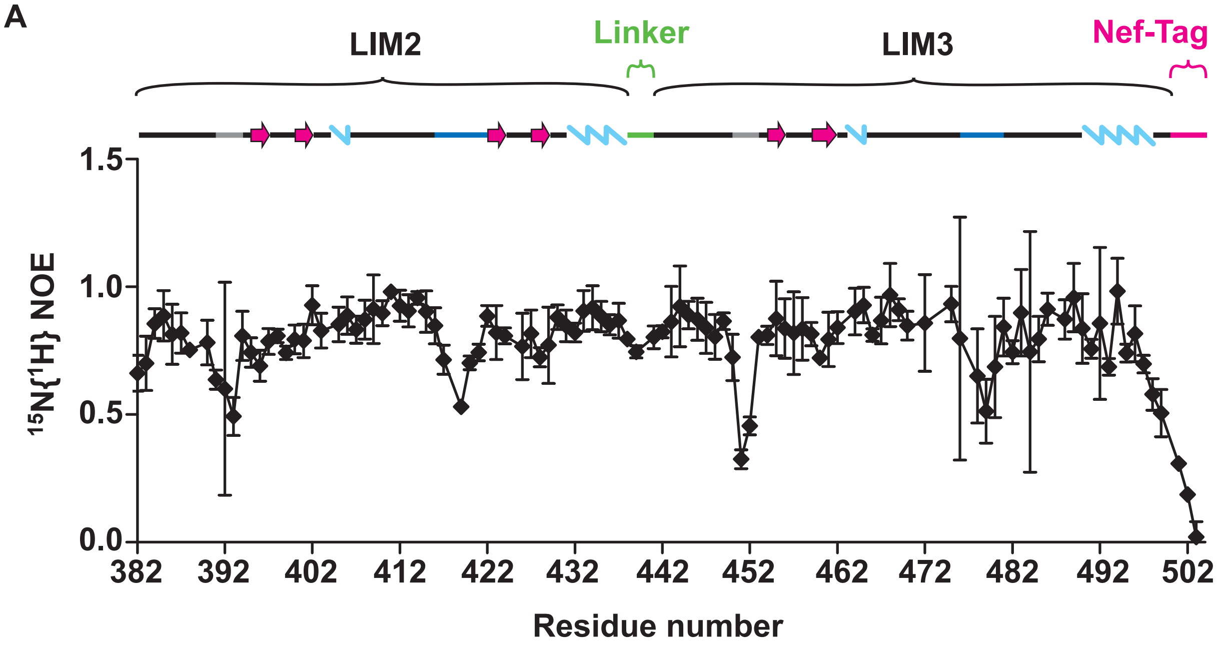

B

```
paxillin, LIM3              440  PKCGGCARAILENYISALNTLWHPECFVCRECFTPFVNGSFFEHDGQPYCEVHYHERRGS  499
Hic-5                       344  PRCQGCQGPILDNYISALSALWHPDCFVCRECFAPFSGGSFFEHEGRPLCENHFHARRGS  403
leupaxin                    268  PKCGGCNRPVLENYLSAMDTVWHPECFVCGDCFTSFSTGSFFELDGRPFCELHYHHRRG-  326
consensus                        PkCgGCqrpiLeNYiSAl tlWHPeCFVCreCFtpFs GSFFEhdGrPyCEvHyH RRGs

Homo sapiens                440  PKCGGCARAILENYISALNTLWHPECFVCRECFTPFVNGSFFEHDGQPYCEVHYHERRGS  499
Mus musculus                474  PKCGGCARAILENYISALNTLWHPECFVCRECFTPFVNGSFFEHDGQPYCEVHYHERRGS  533
Gallus gallus               444  PKCGGCARAILENYISALNTLWHPECFVCRECFTPFINGSFFEHDGQPYCEVH-------  494
Danio rerio                 416  PKCGGCARAILENYISALNSLWHPECFVCRECFTPFVNGSFFEHEGQPYCEAHYHERRGS  475
Drosophila melanogaster     439  PKCNGCNRAIMENYISALNSQWHPDCFVCRDCRQPFQGGSFFDHEGLPYCETHYHAKRGS  498
Caenorhabditis elegans      293  PKCNGCSQPITSNFITALGTHWHPDCFVCQHCGVSFNGASFFEHNGAPLCERHYHESRGS  350
Capsaspora owczarzki        296  PKCGGCDKAIMADCISALGYQWHPNCFVCAECKKGFNGGSFFEHEGKPFCETHYHAQSGS  355
Dictyostelium discoidum     453  -RCGGCNSPIRGECINALGTQWHPEHFVCQYCQKSFTNGQFFEFGGKPYCDVHYHQQAG-  510
consensus                        pkCgGCaraIlenyIsALntlWHPecFVCreCftpFvngsFFeheGqPyCevHyhekrgs

paxillin, LIM2              380  PRCYYCNGPILDKVVTALDRTWHPEHFFCAQCGAFFGPEGFHEKDGKAYCRKDYFDMFA-  439
paxillin, LIM3              440  PKCGGCARAILENYISALNTLWHPECFVCRECFTPFVNGSFFEHDGQPYCEVHYHERRGS  499
consensus                        PrC  C   ILd  vtAL   WHPE F C  C   F    F E DG  YC   Y d  as
```

flexible loop

Supplementary Figure S2: Zinc fingers of the paxillin LIM3 domain show increased structural flexibility and are highly conserved across species

(A) Heteronuclear $^{15}N[^{1}H]$ NOE. The intensity ratio between spectra with and without ^{1}H saturation is displayed vs residue number. Values of 0.8 indicate rigid parts of the structure. Values smaller than 0.8 indicate increasing flexibility on the ps-to-ns timescale. The data underlying this panel can be found in S1_Data.xlsx. On top of the heteronuclear NOE plot, the domain and secondary structure arrangement of the paxillin construct used in this study is depicted. α-Helices are shown in light blue. β-Sheets as magenta arrows. The secondary structure elements depicted here were identified with Pymol's dss command and correspond to the cartoon representation of the 3D structural ensemble shown in Fig. 2. The linker connecting the LIM2 with the LIM3 domain is shown in green. Regions harboring flexible loops are shown in blue and grey, respectively. (B) Sequence alignment of paxillin LIM3 domain across different species: Alignment was performed using the structural alignment tool from T-Coffee and coloured using the BoxShade tool at ExPASy. Identical residues are shaded in black; highly similar residues are shaded gray. In addition, an alignment is shown of paxillin's LIM2 and LIM3 domains. The region harboring a flexible loop which is crucial for binding of the LIM3 domain to b-integrin, is indicated by a blue line.
