# Supplementary material for: A flexible loop in the paxillin LIM3 domain mediates its direct binding to integrin β subunits
Source: PLoS Biol. 2024 Sep 4;22(9):e3002757. doi: 10.1371/journal.pbio.3002757 (PMC11374337; doi:10.1371/journal.pbio.3002757)
Supplement: S3 Fig — (A) 15N-HSQC titration of 300 μM 15N integrin β1 ct (ITGB1 ct) with paxillin LIM2/3 (PXN LIM2/3). Paxillin was added in concentrations up to 500 μM. Boxes show a selection of signals affected by CSPs (residues K784, T788, and T789) in the presence of 0 μM (black), 150 μM (green), 300 μM (blue), and 650 μM (red) paxillin LIM2/3. Insets show the concentration dependence of combined amide CSPs globally fitted to a one site binding model. (B) Combined amide CSPs of 300 μM 15N integrin β1 ct in the presence of 650 μM paxillin LIM2/3 vs. residue number of integrin β1 ct. (C) 15N-HSQC titration of 300 μM 15N paxillin LIM2/3 (PXN LIM2/3) with integrin β3 ct Δ8aa (ITGB3 Δ8aa). Integrin was added up to a concentration of 600 μM. (D) Combined amide CSPs of 300 μM 15N paxillin LIM2/3 in the presence of 600 μM integrin β3 ct Δ8aa vs. residue number of paxillin LIM2/3. (E) 293T cells were transiently cotransfected with a CEACAM3 ITGB3 (CEA3-ITGB3) fusion construct or the indicated truncated ITGB3 mutants together with GFP or GFP-paxillin and seeded on poly-L-lysine. Cells were infected for 1 h with Pacific Blue–labelled Neisseria gonorrhoeae (Ngo, blue), fixed, and stained for ITGB3 (red). Recruitment of GFP-Paxillin to clustered ITGB3 tails is indicated by white arrowheads. Bars represent 1 μm. (F) Quantification of GFP/GFP-paxillin recruitment to the indicated CEA3-ITGB3 variants from (E). Shown are means and 95% confidence intervals of n = 60 cells from 3 independent experiments. Significance was calculated using one-way ANOVA followed by Bonferroni multiple comparison test. Significance levels compared to paxillin wt are indicated (ns: not significant; *** p ≤ 0.001). The data underlying this panel can be found in S1 Data. (G) Streptactin pulldown of recombinant His-SUMO or His-SUMO-talin F3 using the Strep-tag integrin β3 cytoplasmic tail in the wt form or with a truncation of the carboxy-terminal 3 (Δ3) or 8 (Δ8) amino acids. Integrin β3–associated talin F3 domain was detec [file pbio.3002757.s003.pdf]

Baade et al., Suppl. Fig. S3

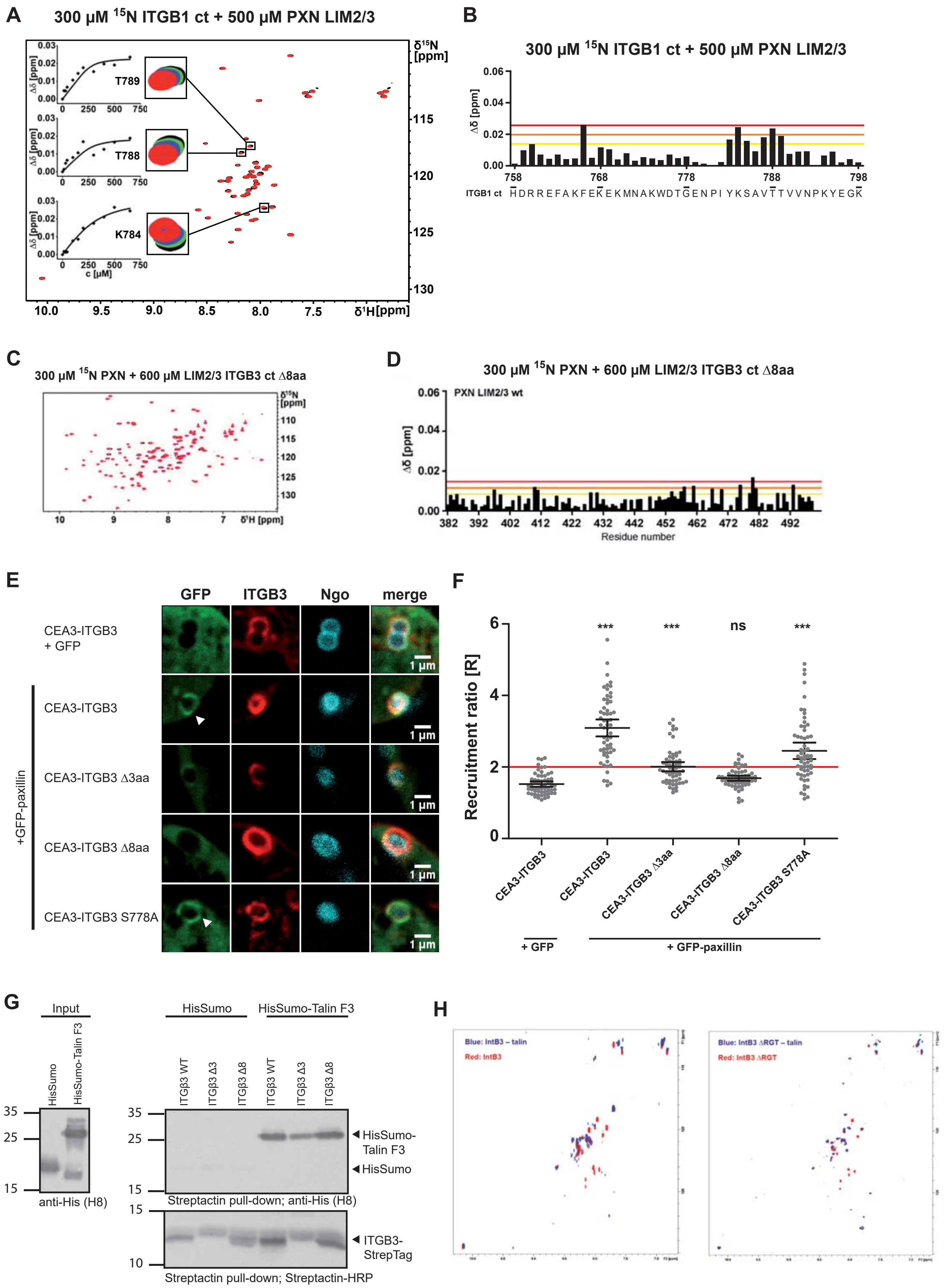

Supplementary Figure S3: The cytoplasmic domains of integrin-β1 and integrin-β3 support direct binding of paxillin.

(A) ^{15}N-HSQC titration of 300 µM ^{15}N integrin β1 ct (ITGB1 ct) with paxillin LIM2/3 (PXN LIM2/3). Paxillin was added in concentrations up to 500 µM. Boxes show a selection of signals affected by CSPs (residues K784, T788 & T789) in the presence of 0 µM (black), 150 µM (green), 300 µM (blue) and 650 µM (red) paxillin LIM2/3. Insets show the concentration dependence of combined amide CSPs globally fitted to a one site binding model. (B) Combined amide CSPs of 300 µM ^{15}N integrin β1 ct in the presence of 650 µM paxillin LIM2/3 vs residue number of integrin β1 ct. (C) ^{15}N-HSQC titration of 300 µM ^{15}N paxillin LIM2/3 (PXN LIM2/3) with integrin β3 ct Δ8aa (ITGB3 Δ8aa). Integrin was added up to a concentration of 600 µM. (D) Combined amide CSPs of 300 µM ^{15}N paxillin LIM2/3 in the presence of 600 µM integrin β3 ct Δ8aa vs residue number of paxillin LIM2/3. (E) 293T cells were transiently co-transfected with a CEACAM3 ITGB3 (CEA3-ITGB3) fusion construct or the indicated truncated ITGB3 mutants together with GFP or GFP-paxillin and seeded on poly-L-lysine. Cells were infected for 1 h with Pacific Blue-labelled *Neisseria gonorrhoeae* (Ngo, blue), fixed, and stained for ITGB3 (red). Recruitment of GFP-Paxillin to clustered ITGB3 tails is indicated by white arrowheads. Bars represent 1 µm. (F) Quantification of GFP/GFP-paxillin recruitment to the indicated CEA3-ITGB3 variants from (E). Shown are means and 95% confidence intervals of n=60 cells from three independent experiments. Significance was calculated using one-way ANOVA followed by Bonferroni Multiple Comparison Test. Significance levels compared to paxillin wt are indicated (ns: not significant; *** $p \leq 0.001$). The data underlying this panel can be found in S1_Data.xlsx. (G) Streptactin pulldown of recombinant His-SUMO or His-SUMO-talin F3 using the Strep-tag integrin β3 cytoplasmic tail in the

wildtype form (wt) or with a truncation of the carboxy-terminal 3 (Δ3) or 8 (Δ8) amino acids. Integrin b3-associated talin F3 domain was detected by anti-His-tag blot (upper panel), the Strep-tag integrin tails were revealed by streptactin blot (lower panel). The input of purified His-SUMO or His-SUMO-Talin F3 protein is shown on the left hand side. (H) NMR-based interaction study between talin‘s FERM domain and integrin- β3. Superposition of ^{15}N-HSQC spectra of ^{15}N-labeled wildtype ITGB3 (left graph) or ITGB3 Δ3 (ΔRGT; right graph). In both experiments, multiple signals of the integrin peptide shift or disappear completely through the interaction with the large FERM domain indicating equivalent binding of the wildtype and Δ3 integrin peptide to talin.
