# Supplementary material for: A flexible loop in the paxillin LIM3 domain mediates its direct binding to integrin β subunits
Source: PLoS Biol. 2024 Sep 4;22(9):e3002757. doi: 10.1371/journal.pbio.3002757 (PMC11374337; doi:10.1371/journal.pbio.3002757)
Supplement: S4 Fig — Alanine scan of paxillin LIM3’s flexible loop region. Shown are superpositions of 1H-15N-HSQC spectra of wt paxillin LIM2/3 (black) and paxillin LIM2/3 mutants (red). The bar graphs below show the combined amide CSPs of 15N-labelled paxillin LIM2/3 wt compared to the indicated mutant paxillin LIM2/3 along the amino acid sequence. (A) Paxillin LIM2/3 F475A. (B) Paxillin LIM2/3 F480A. (C) Paxillin LIM2/3 F481A. (D) Paxillin LIM2/3 V476A. (E) Paxillin LIM2/3 S479A. (F) Paxillin LIM2/3 4A. (PDF) [file pbio.3002757.s004.pdf]

Baade et al., Suppl. Fig. S4

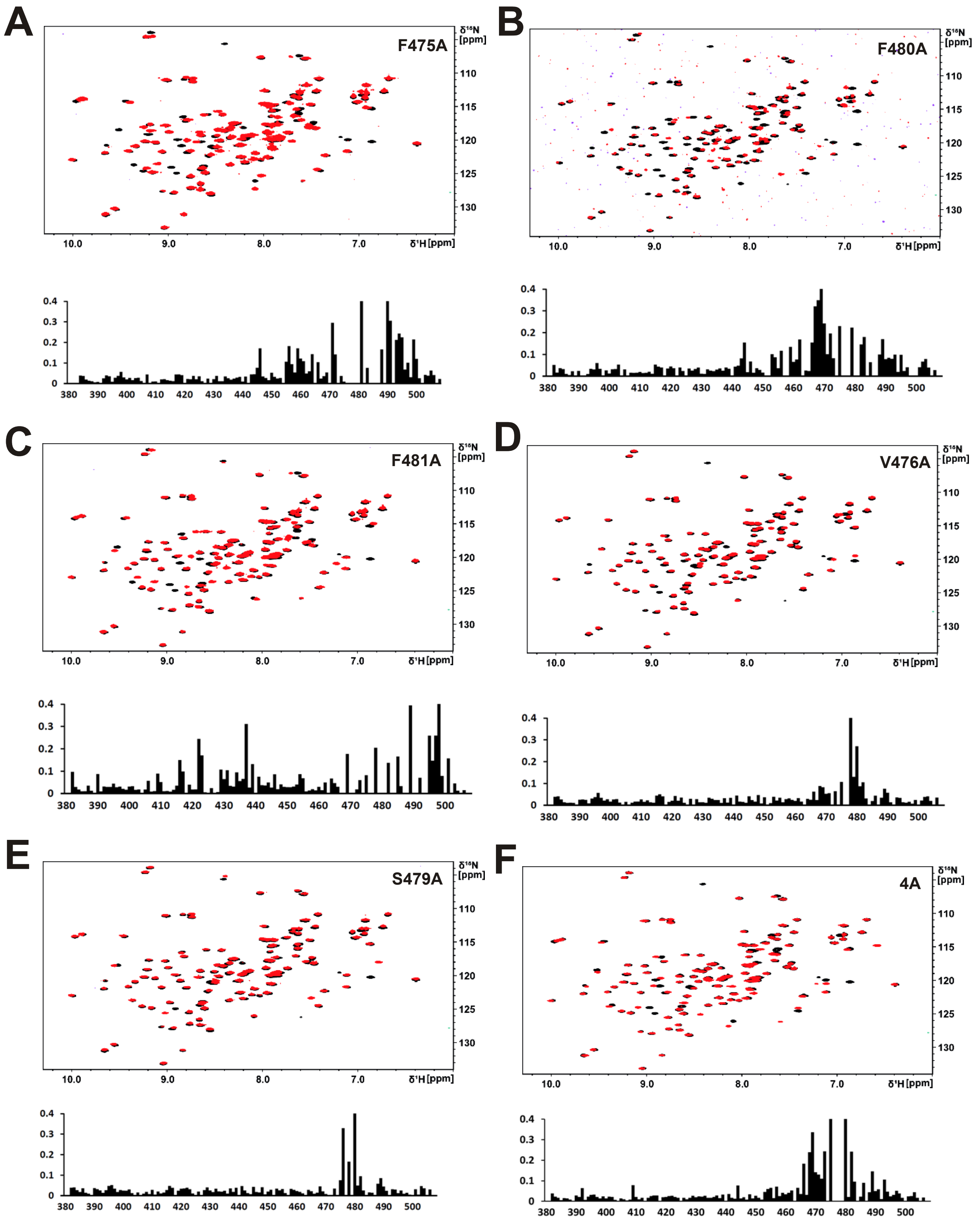

Supplementary Figure S4: NMR experiments with paxillin LIM3 mutants show that conserved aromatic residues in the flexible loop are essential for maintaining a stably folded structure.

Alanine scan of paxillin LIM3's flexible loop region. Shown are superpositions of ^{1}H-^{15}N-HSQC spectra of wildtype paxillin LIM2/3 (black) and paxillin LIM2/3 mutants (red). The bar graphs below show the combined amide CSs of ^{15}N-labelled paxillin LIM2/3 wildtype compared to the indicated mutant paxillin LIM2/3 along the amino acid sequence. (A) Paxillin LIM2/3 F475A. (B) Paxillin LIM2/3 F480A. (C) Paxillin LIM2/3 F481A. (D) Paxillin LIM2/3 V476A. (E) Paxillin LIM2/3 S479A. (F) Paxillin LIM2/3 4A.
