# Supplementary material for: A flexible loop in the paxillin LIM3 domain mediates its direct binding to integrin β subunits
Source: PLoS Biol. 2024 Sep 4;22(9):e3002757. doi: 10.1371/journal.pbio.3002757 (PMC11374337; doi:10.1371/journal.pbio.3002757)
Supplement: S5 Fig — (A) NIH 3T3 Flp-In cells (NIH3T3) were used to derive paxillin KO cells. Paxillin KO cells were stably transfected with the empty vector (PXN KO) or vectors encoding GFP-paxillin wt (PXN wt), paxillin lacking the LIM4 domain (PXN ΔLIM4), or paxillin with a mutated flexible loop in LIM3 (PXN-4A). Western blot of WCLs with anti-paxillin antibody demonstrates the lack of endogenous paxillin in the PXN KO cells and reexpression of equivalent levels of paxillin wt or mutants in the stably reexpressing cells (upper panel). The lower panel verifies equal loading of samples by anti-tubulin blot. (B) Cells from (A) were starved overnight and seeded for 30 or 120 min, respectively, on the integrin ligand fibronectin in the absence of serum. Cells were fixed and the cell membrane was stained with CellMask Orange. Scale bar represents 20 μm (upper panel). To analyse cell spreading, the area of individual cells was quantified (lower panel). Shown are mean values with 95% confidence intervals from 3 independent experiments for 30-min time point or from 2 independent experiments for 120-min time point. Sample sizes are given in brackets. Statistical significance was calculated using one-way ANOVA followed by Bonferroni multiple comparison test (ns: not significant; *** p ≤ 0.001; ** p ≤ 0.01). The data underlying this panel can be found in S1 Data. (C) TIRF microscopy images of PXN KO and reexpressing cell lines seeded on vitronectin for 30 min before fixation. Cells were stained with a monoclonal anti-kindlin antibody. (D, E) FA analysis of n > 1,000 kindlin-positive FAs per sample. FAs were analysed for GFP-paxillin intensity (D) and FA area (E). Violin plot in (E) show the Kernel probability density distribution. Densities are plotted symmetrically to the left and right of the box plots. Inserted boxplots show mean values with 95% confidence intervals. Statistical significance was calculated using one-way ANOVA followed by Bonferroni multiple comparison test. Significance level [file pbio.3002757.s005.pdf]

Baade et al., Suppl. Fig. S5

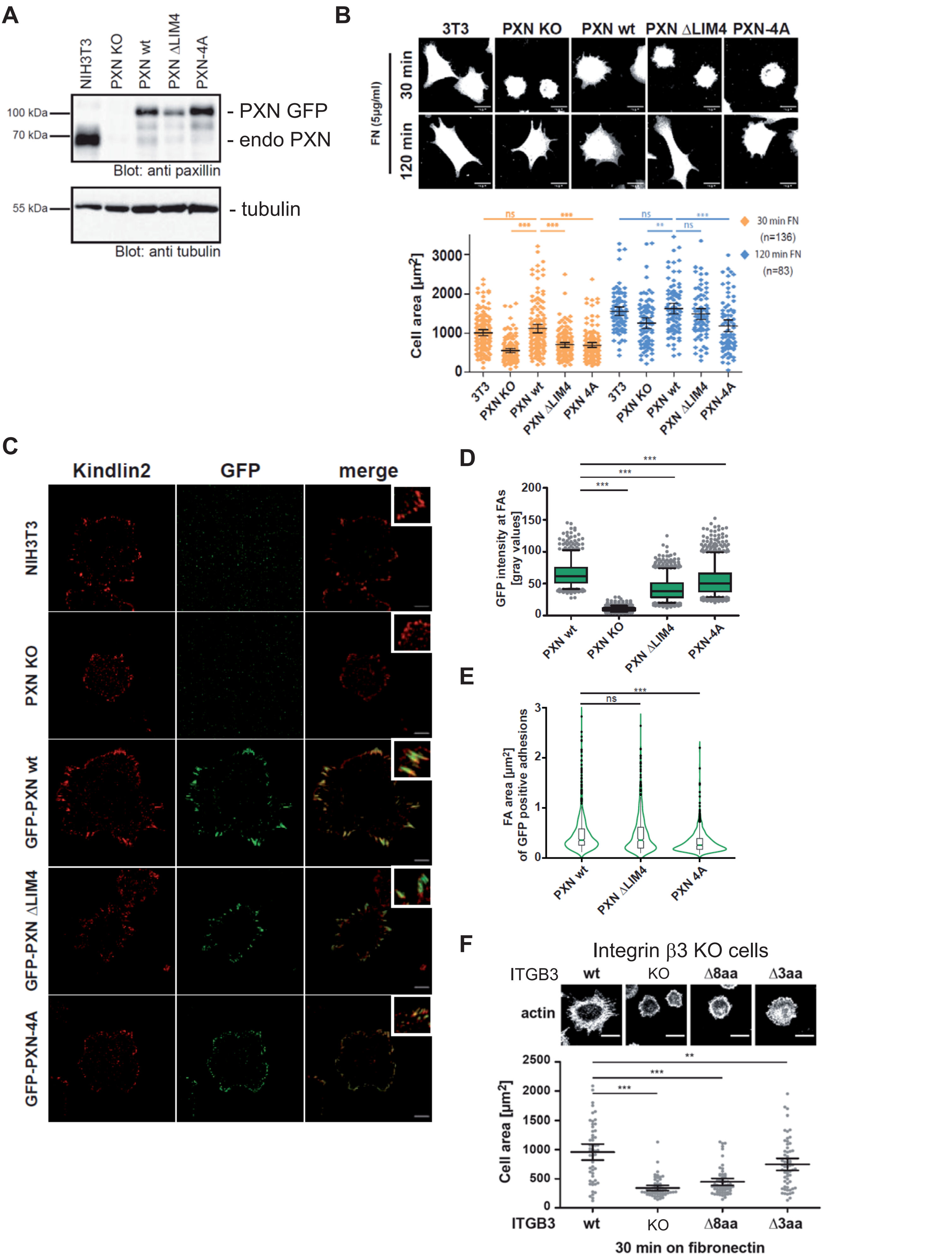

Supplementary Figure S5: The direct interaction between the paxillin LIM3 flexible loop and integrin β3 contributes to cell spreading.

(A) NIH 3T3 Flp-In cells (NIH3T3) were used to derive paxillin knockout cells. Paxillin KO cells were stably transfected with the empty vector (PXN KO) or vectors encoding GFP-paxillin wt (PXN wt), paxillin lacking the LIM4 domain (PXN ΔLIM4), or paxillin with a mutated flexible loop in LIM3 (PXN-4A). Western Blot of whole cell lysates with anti-paxillin antibody demonstrates the lack of endogenous paxillin in the PXN KO cells and re-expression of equivalent levels of paxillin wt or mutants in the stably re-expressing cells (upper panel). The lower panel verifies equal loading of samples by anti-tubulin blot. (B) Cells from (A) were starved overnight and seeded for 30 or 120 min, respectively on the integrin ligand fibronectin in the absence of serum. Cells were fixed and the cell membrane was stained with CellMask Orange. Scale bar represents 20 µm. (upper panel). To analyse cell spreading, the area of individual cells was quantified (lower panel). Shown are mean values with 95% confidence intervals from 3 independent experiments for 30 min timepoint or from 2 independent experiments for 120 min timepoint. Sample sizes are given in brackets. Statistical significance was calculated using one-way ANOVA followed by Bonferroni Multiple Comparison Test (ns: not significant; *** $p \leq 0.001$; ** $p \leq 0.01$). The data underlying this panel can be found in S1_Data.xlsx. (C) TIRF microscopy images of PXN KO and re-expressing cell lines seeded on vitronectin for 30 min before fixation. Cells were stained with a monoclonal anti-kindlin antibody. (D) and (E) Focal adhesion analysis of n>1000 kindlin-positive FAs per sample. FAs were analysed for GFP-paxillin intensity (D) and FA area (E). Violin plot in (E) show the Kernel probability density distribution. Densities are plotted symmetrically to the left and right of the box plots. Inserted box-plots show mean values with 95% confidence intervals. Statistical significance was calculated

using one-way ANOVA followed by Bonferroni Multiple Comparison Test. Significance levels compared to paxillin wt are indicated (ns: not significant; *** $p \leq 0.001$; ** $p \leq 0.01$; * $p \leq 0.05$). Box plots in (D) show mean and error bars represent 5 and 95 percentiles. Significance was calculated using one-way ANOVA, followed by Bonferroni Multiple Comparison Test (*** $p < 0.001$, ns = not significant). (F) NIH Flp-In integrin β3 knockout (KO) and the indicated ITGB3 wt, ITGB3 Δ8aa, or ITGB3 Δ3aa re-expressing cell lines were serum starved and seeded onto fibronectin-coated (5 µg/ml) glass slides for 30 min and cell area was measured. Shown are mean values and 95% confidence intervals of n=60 cells per sample from 3 independent experiments. Statistical significance was calculated using one-way ANOVA followed by Bonferroni Multiple Comparison Test (*** $p \leq 0.001$; ** $p \leq 0.01$). The data underlying panels D) – F) can be found in S1_Data.xlsx.
