# Supplementary material for: A flexible loop in the paxillin LIM3 domain mediates its direct binding to integrin β subunits
Source: PLoS Biol. 2024 Sep 4;22(9):e3002757. doi: 10.1371/journal.pbio.3002757 (PMC11374337; doi:10.1371/journal.pbio.3002757)
Supplement: S1 Raw Images — (PDF) [file pbio.3002757.s007.pdf]

Figure 1F

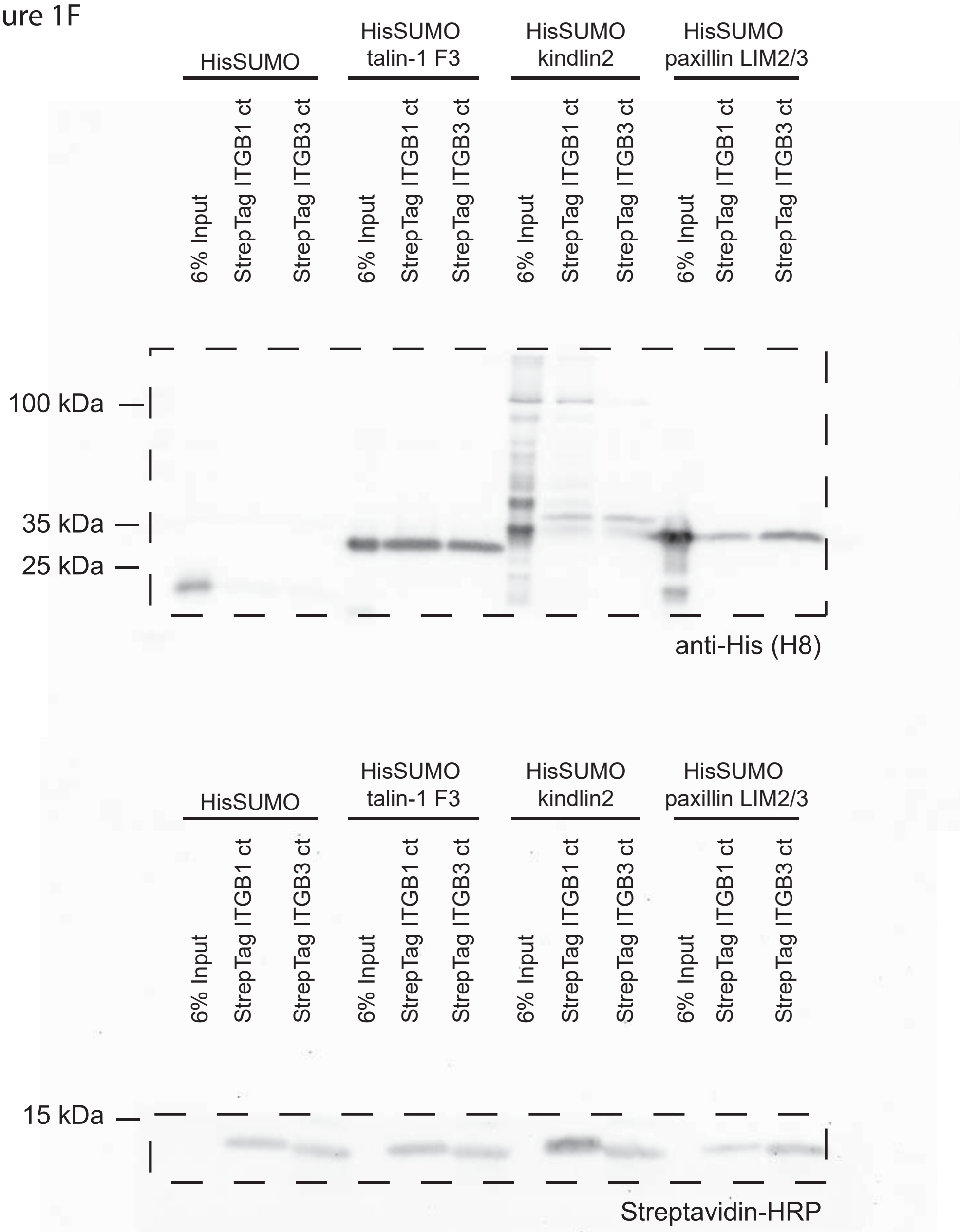

Unmodified original image of blot shown in Fig.1F.
Whole blot has been uniformly enhanced in brightness and contrast for better visibility.
Membrane was cut ~18 kDa to probe resulting top and bottom membrane parts
with different antibodies (top: anti-His; bottom: strep-HRP).

Figure 2F

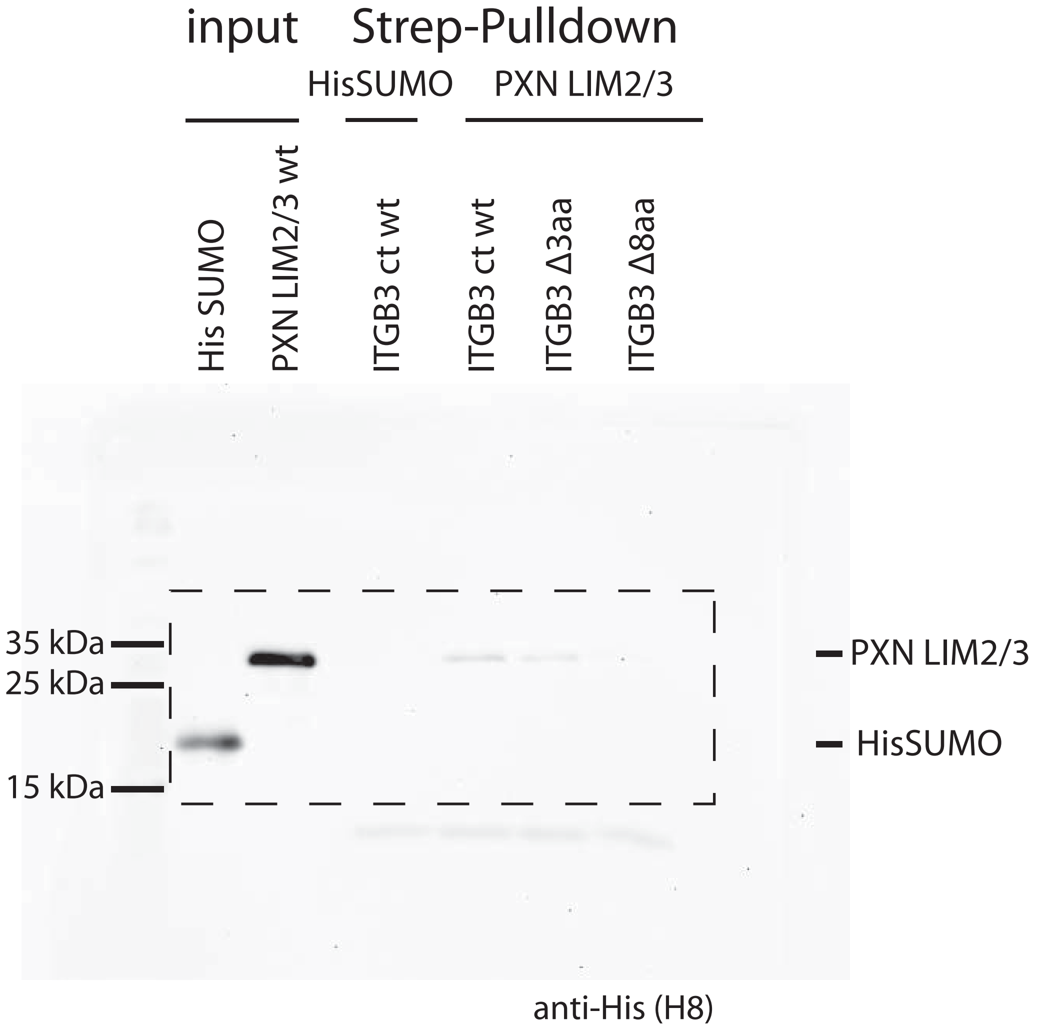

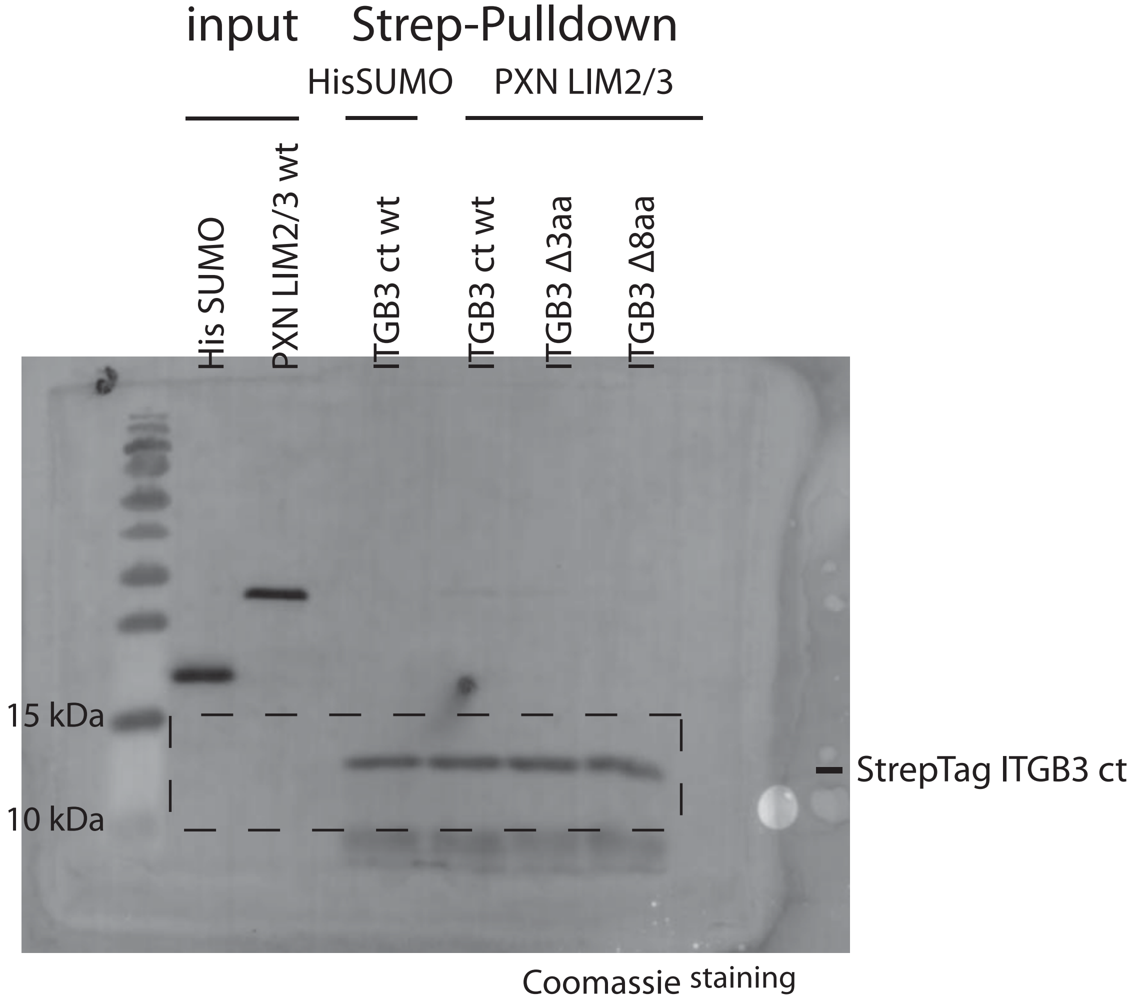

Unmodified original image of blot shown in Fig.2F. Blot shown in manuscript has been uniformly enhanced in brightness and contrast for better visibility. Lower panel shows Coomassie staining of the membrane visualizing protein standard and pulled-down StrepTag ITGB3 ct

Figure 3E

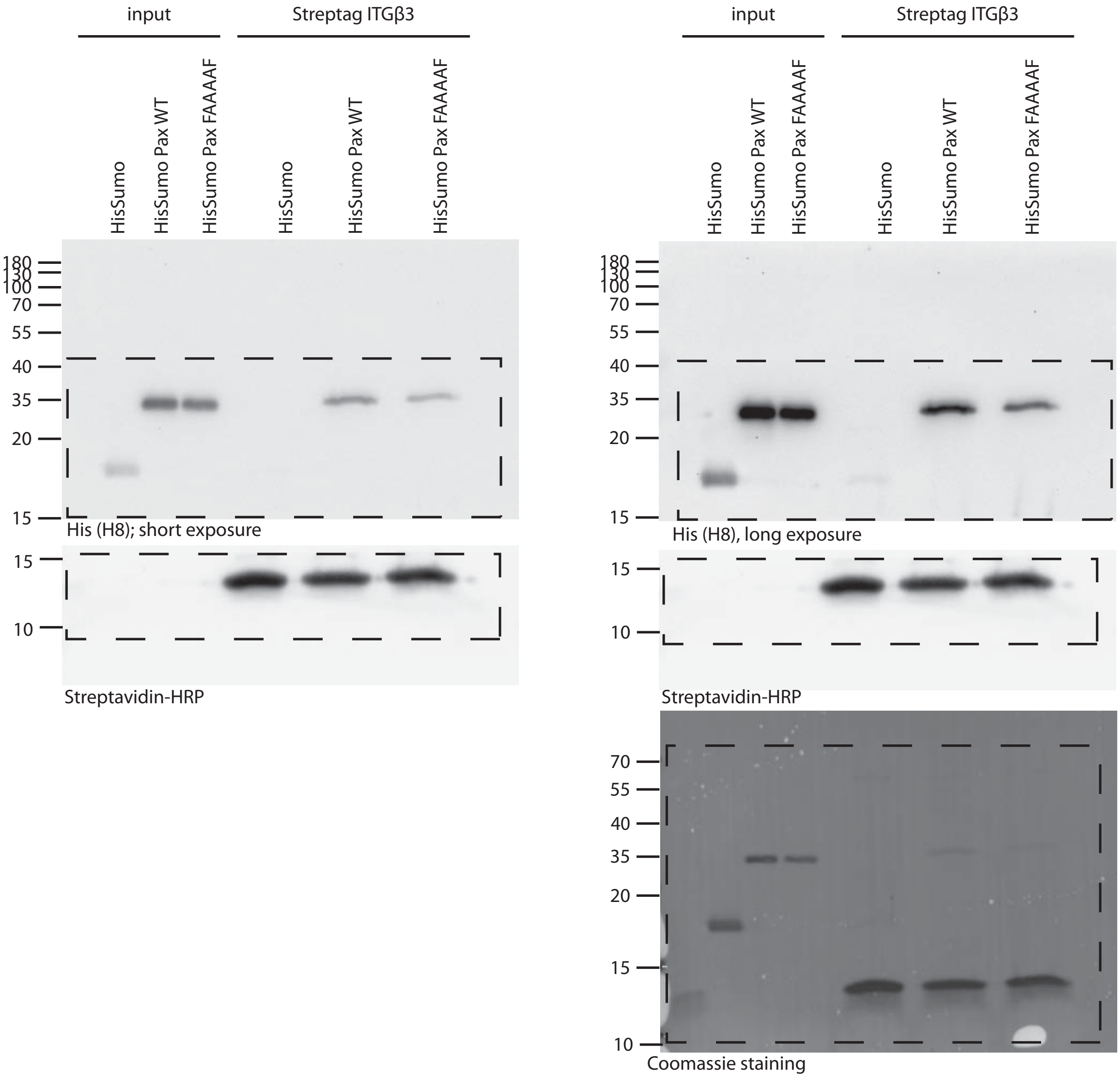

Unmodified original images of blots shown in Fig.3E. Blot shown in manuscript has been uniformly enhanced in brightness and contrast for better visibility. Membrane was cut ~15 kDa to probe resulting top and bottom membrane parts with different antibodies (top: anti-His; bottom: strep-HRP).
Lower panel shows Coomassie staining of the membrane.

Figure 4B

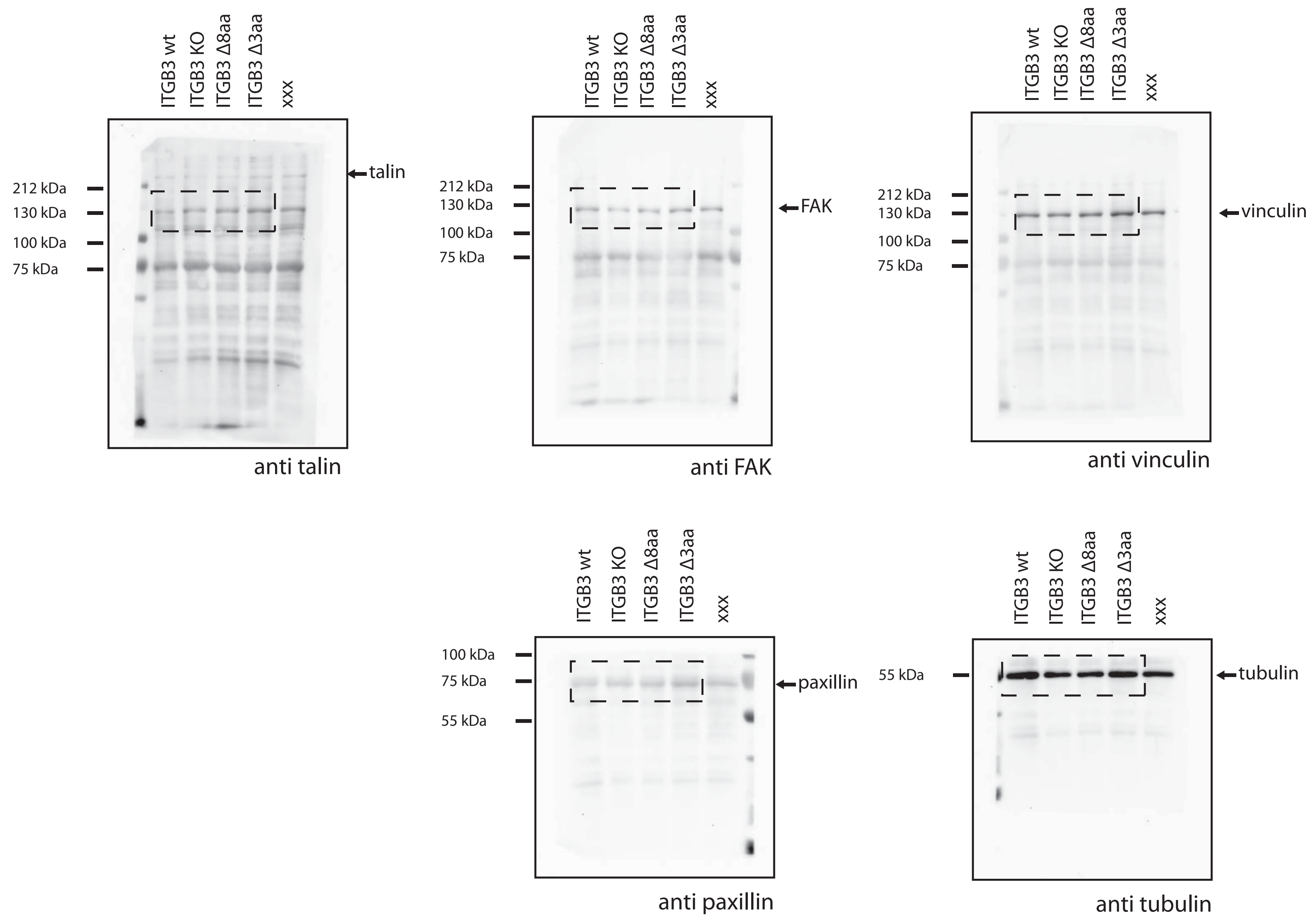

Unmodified original images of blots shown in Fig.4B. Blots shown in manuscript have been uniformly enhanced in brightness and contrast for better visibility. xxx=irrelevant cell line not used in the manuscript

Figure 4E

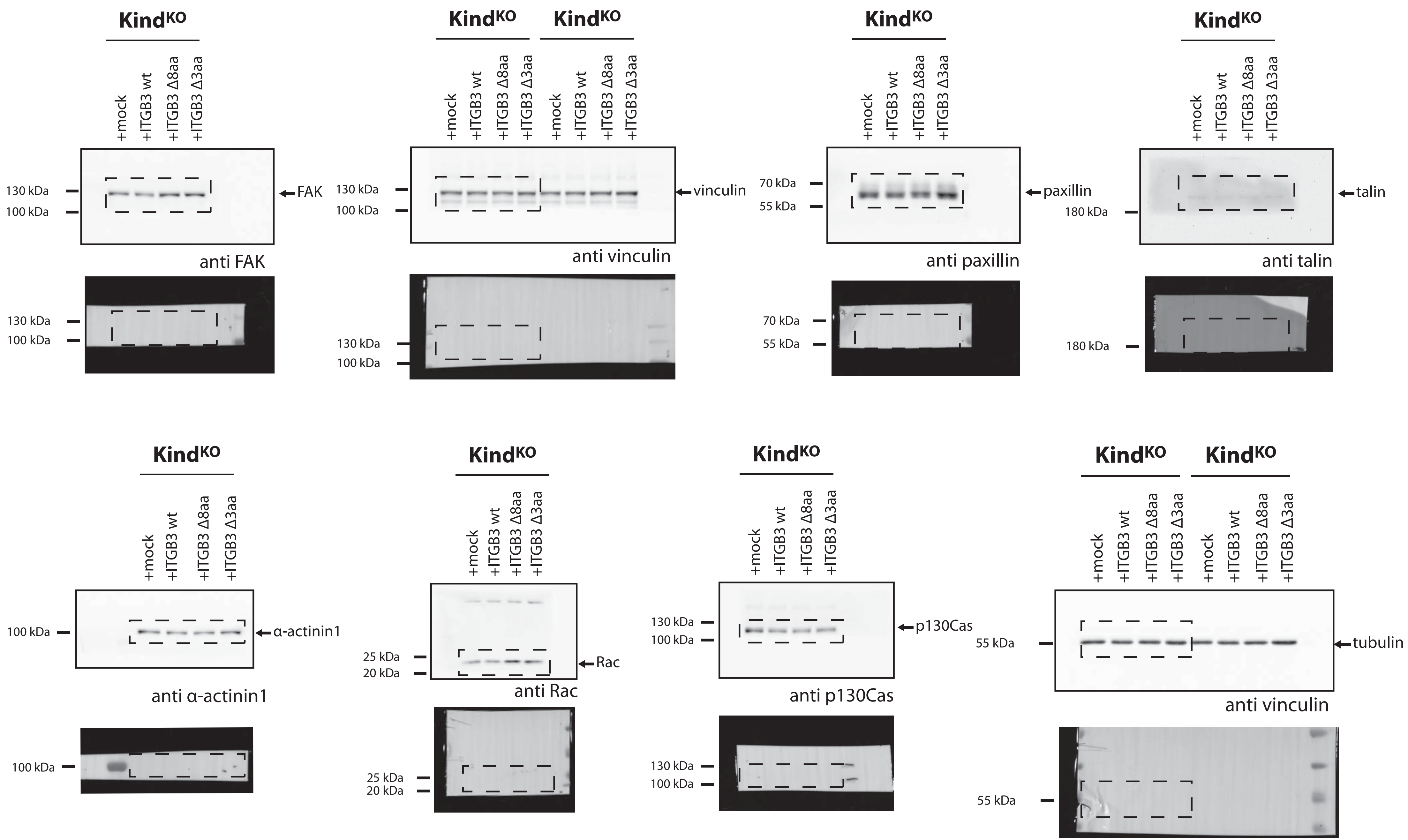

Unmodified original images of blots shown in Fig.4E . Blots shown in manuscript have been uniformly enhanced in brightness and contrast for better visibility. Blots generated in duplicates and cut according to expected protein size for simultaneous processing with multiple antibodies

Figure S1B

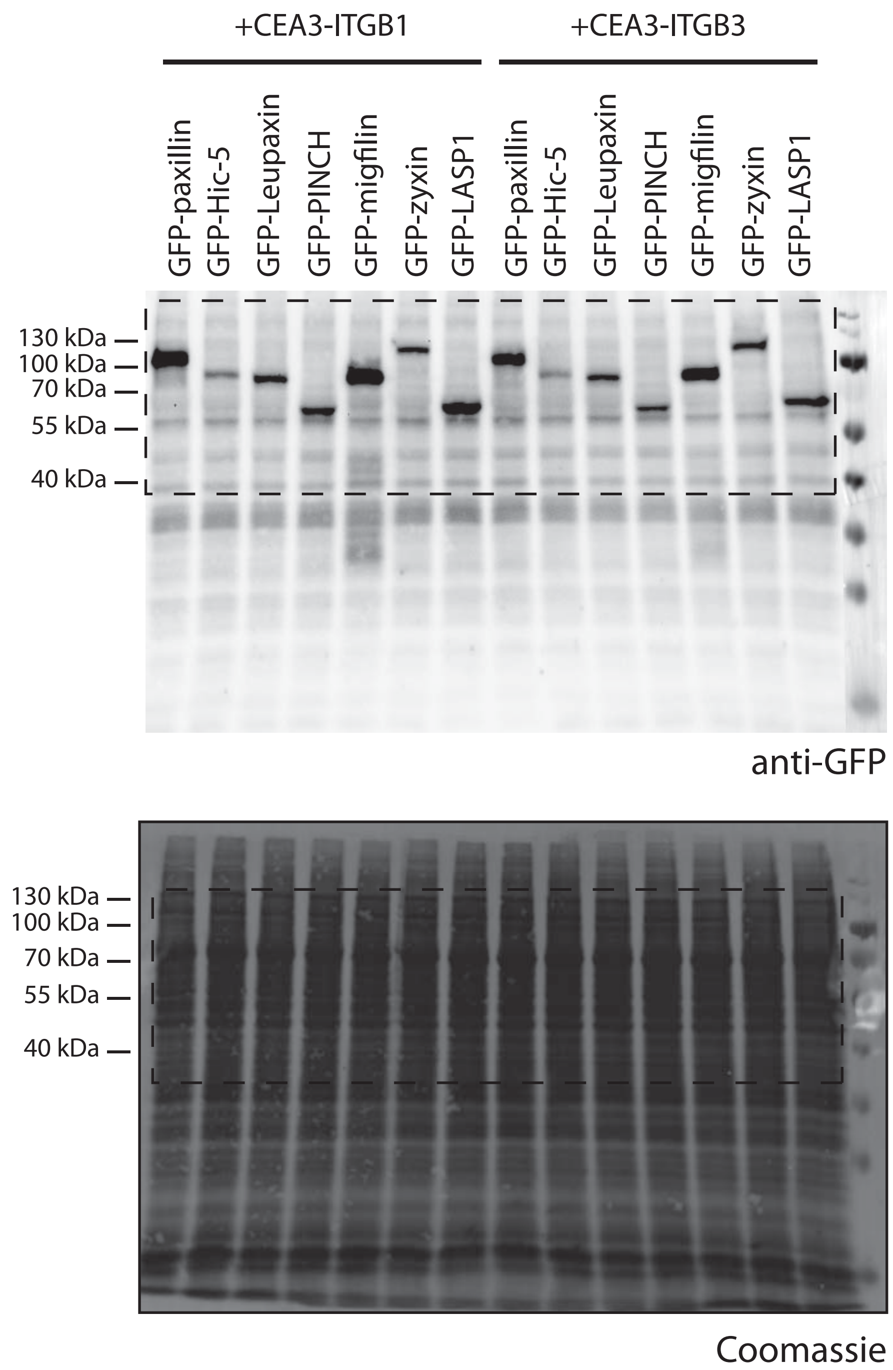

Unmodified original images of blots shown in Fig.4E . Blots shown in manuscript have been uniformly enhanced in brightness and contrast for better visibility.

Supplemental Figure S3G

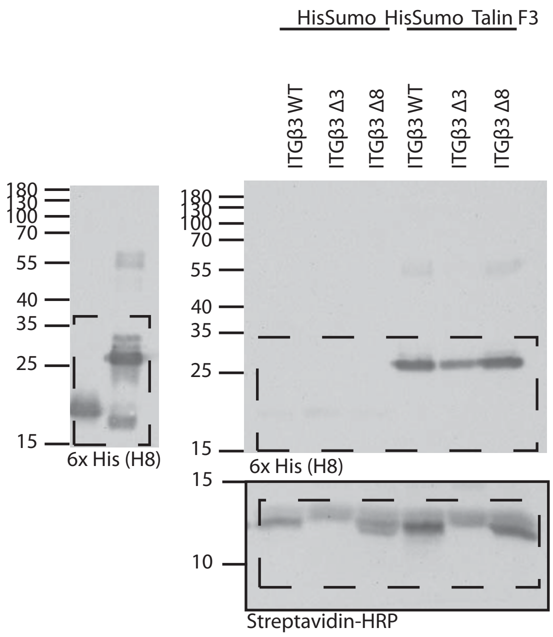

Unmodified original images of blots shown in Fig. S3G. Blot shown in manuscript has been uniformly enhanced in brightness and contrast for better visibility. Membrane on the right side was cut ~15 kDa to probe resulting top and bottom membrane parts with different antibodies (top: anti-His; bottom: strep-HRP).
Lower panel shows Coomassie staining of the membrane.

Figure S5A

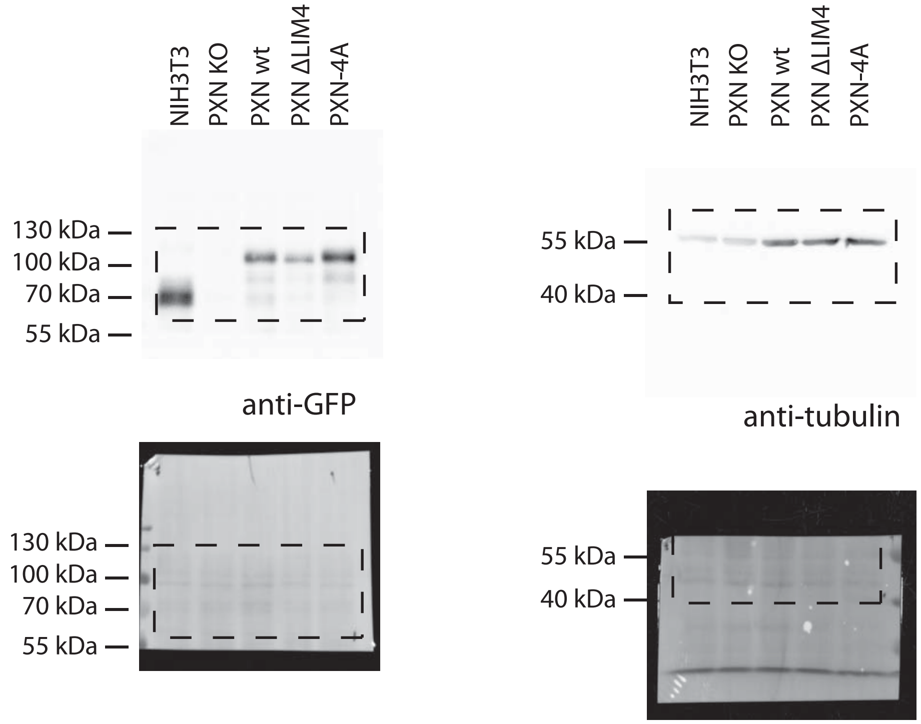

Unmodified original images of blots shown in Fig.S5A . Blots shown in manuscript have been uniformly enhanced in brightness and contrast for better visibility.
