# Supplementary material for: A flexible loop in the paxillin LIM3 domain mediates its direct binding to integrin β subunits
Source: PLoS Biol. 2024 Sep 4;22(9):e3002757. doi: 10.1371/journal.pbio.3002757 (PMC11374337; doi:10.1371/journal.pbio.3002757)
Supplement: Attachment — Submitted filename: Baade_et_al-Response_to_Reviewers.pdf [file pbio.3002757.s008.pdf]

REVIEWS:

Reviewer #1: This manuscript reports the NMR-based structural and functional studies of paxillin LIM2-3 domain and its interaction with integrin beta cytoplasmic tails. The major conclusion is that such interaction is crucial for recruiting paxillin to the integrin adhesion site independent of kindlin. While the interaction appears to be novel, the physiological relevance of this interaction and the authors' major conclusion are not supported by the data. It has been well established that talin and kindlin, both of which bind to paxillin as the authors indicated, directly bind to integrin and trigger focal adhesion assembly. In fact, it was shown clearly that kindlin, which binds paxillin at nM affinity (Böttcher RT et al., J Cell Biol, 2017) and recruits paxillin to focal adhesion (Theodosiou et al., eLife, 2016). Of course, talin-paxillin interaction also promotes paxillin recruitment to integrin site. These earlier data show clearly that paxillin coordinates with kindlin and talin to be recruited to the integrin site not independently contrasting to what the authors proposed here. The technical flaws I saw in the authors' experiment are:

1. The paxillin LIM3 binding site on integrin tail overlaps with that of kindlin and probably part of talin too (Fig 2D) based on the talin-integrin and kindlin-integrin complex structures. Thus, the 3aa and 8aa deletion in integrin tail would clearly affect kindlin and likely talin too. These deletion data cannot support the paxillin recruitment to integrin site independent of kindlin and likely talin.

We thank the reviewer for this comment, which is well taken. As mentioned by the reviewer, previous reports have identified a direct interaction between kindlin F0/PH domains and the paxillin LD motifs and/or the LIM4 domain of paxillin (Böttcher RT et al. (2017) J Cell Biol, 216: 3785-3798; Theodosiou et al. (2016) eLife, 5: e10130) and also between talin R8 and paxillin LD motifs (Zacharchenko T et al. (2016) Structure. 24: 1130-41. doi: 10.1016/j.str.2016.04.016).

We totally agree with these prior studies and acknowledge that paxillin can be indirectly connected via kindlin and/or talin to the integrin beta cytoplasmic tail. However, our data also demonstrate that in addition to these known indirect connections, paxillin LIM2/3 domains can also directly bind to integrin beta tails. In particular we show for the integrin beta3 subunit, that this direct association with paxillin occurs in the absence of kindlin and talin (as seen by all the biochemical interaction studies with recombinant proteins and/or synthetic peptides presented in Fig. 1F, Fig. 2, and Fig. 3) and that this direct association of paxillin requires the C-terminal amino acid residues of the ITGB3 subunit (Fig. 2). Most importantly, we can demonstrate that this direct association of paxillin with integrin beta 3 allows paxillin recruitment to vitronectin-based cell attachment sites in murine fibroblasts devoid of kindlin1 and kindlin2 (Fig. 4F). This finding is in line with the results of Theodosiou et al. (2016, eLife, 5: e10130), which also reported robust recruitment of Paxillin to mature focal adhesions in Kindlin1&2 knock-out cells re-expressing a truncated Kindlin2 version, which lacks paxillin binding (ΔPH-Kindlin2).

Theodosiou et al. conclude in their discussion: “*The formation of paxillin-positive FAs in cells expressing the PH domain-deficient kindlin-2 suggests that the recruitment of paxillin to FAs occurs either in a kindlin-independent manner or through a modification of kindlin in a second binding motif.*” We believe that our data nicely confirm the speculation of Theodosiou et al. and provide a molecular explanation for the kindlin-independent recruitment of paxillin via a direct connection to the integrin beta tail.

The reviewer correctly points to the potential role of talin in recruiting paxillin to the integrin beta 3 tail in these kindlin1/2-knock-out cells, as the talin R8 domain is able to interact with paxillin LD motifs (Zacharchenko T et al. (2016) Structure. 24: 1130-41. doi: 10.1016/j.str.2016.04.016). However, a talin-mediated indirect recruitment would not explain the lack of paxillin-positive attachment sites in kindlin1/2-deficient cells, which express the truncated integrin beta3 variants (ITGB3 Δ8 and ITGB3 Δ3). Indeed, these C-terminal truncations of integrin beta 3 do not compromise the talin-integrin interaction, but abolish paxillin binding. To clearly demonstrate this fact, we have now conducted additional biochemical binding studies with the recombinant talin F3 domain and the integrin beta3 cytoplasmic domain, either in the full-length form or in the truncated versions (ITGB3 Δ8 and ITGB3 Δ3). As can be seen in the novel Suppl. Fig. S3G, pull-down assays demonstrate that while the paxillin LIM2/3 domain looses binding to the truncated ITGB3 tails (Fig. 2A), the binding of the talin F3 domain is unaffected. These pull-down assays are further corroborated by NMR measurements which suggest equivalent interaction between the talin head domain and ITGB3 wildtype as well as ITGB Δ3 tails. Accordingly, we conclude that the recruitment of paxillin to the integrin beta3 tail in intact cells is supported by the direct interaction of paxillin LIM2/LIM3 with the C-terminal amino acids of the integrin tail, which we characterize biochemically. This direct interaction does not require the presence of kindlin and it cannot be compensated by the presence of talin at the integrin tail, as paxillin recruitment is lost, when the C-terminal amino acids of integrin beta3 are truncated, which does not compromise talin binding.

2. The mutations on LIM3 loop caused some cellular defects. However, given that LIM3 may also partially bind to other proteins such as Kindlin (Böttcher RT et al., J Cell Biol, 2017) and PTP-PEST (Cote et al., J. Biol. Chem, 274:20550-60, 1999), it is unclear if the mutations would also impair the interaction with these proteins, making the analysis ambiguous.

The reviewer cautions that mutations introduced in the flexible loop of paxillin’s LIM3 domain, which we identify as a region involved in integrin tail binding, might also corrupt other paxillin interactions. However, though the study by Böttcher RT et al. refers to the Paxillin LIM3 domain as an interaction site, the cross-links detected in that study by MS analysis connect paxillin K540, which is located in paxillin’s LIM4 domain, with Kindlin (see Suppl. Data file “High-confidence lysine–lysine cross-links of the kindlin-2–paxillin complex.xlsx” in Böttcher et al.). Therefore, the data of Böttcher et al. identify Paxillin LIM4 domain interactions with Kindlin, while our data connect Paxillin LIM2/LIM3 with the integrin tail. Accordingly, the mutations

introduced by us in the flexible loop (Paxillin residues F438-K441) are clearly distant from the K540 residue in paxillin's LIM4 domain reported by Böttcher and colleagues.
Of course there are additional known and potential unknown interactions mediated by the paxillin LIM3 domain that could be corrupted by these mutations. One of the known interactions supported by Paxillin LIM3 is the direct association of paxillin LIM3 and LIM4 with PTP-PEST (Cote et al., J. Biol. Chem, 274:20550-60, 1999). However, as PTP-PEST does not localize to focal adhesions (Shen Y, Schneider G, Cloutier JF, Veillette A, Schaller MD. Direct association of protein-tyrosine phosphatase PTP-PEST with paxillin. J Biol Chem. 1998 Mar 13;273(11):6474-81. doi: 10.1074/jbc.273.11.6474) and as PTP-PEST knock-out fibroblasts show increased, not reduced spreading on extracellular matrix substrates (Angers-Loustau, A., J. Cote, A. Charest, D. Dowbenko, S. Spencer, L.A. Lasky, and M.L. Tremblay. 1999. Protein Tyrosine Phosphatase-PEST Regulates Focal Adhesion Disassembly, Migration, and Cytokinesis in Fibroblasts. J Cell Biol 144:1019-1031.) a potentially disrupted association with this tyrosine phosphatase would not be relevant for recruiting paxillin to the integrin tail, would also not explain the lack of paxillin recruitment to integrin beta 3 mutants as discussed above (see reviewer 1; comment 1), and most importantly, would not explain the phenotype of cells expressing the paxillin LIM3 4A mutant, which shows reduced, but not enhanced spreading.

Together, we cannot rule out that mutations introduced into the flexible loop of paxillin's LIM3 domain might also interfere with the binding of other, currently uncharacterized interaction partners of paxillin. However, given the agreement between paxillin's role in integrin-initiated cell spreading on the extracellular matrix, the biochemical evidence of a direct interaction between paxillin LIM3 and the integrin beta 3 tail, and the small, but clear spreading phenotype of cells expressing the paxillin 4A mutant, we believe that there is in addition to the biochemical data a suite of experimental evidence presented with intact cells (paxillin-knock-out cells re-expressing paxillin mutants and kindlin 1&2-knock-out cells with their integrin beta3-deficiency, expressing integrin beta 3 variants) to suggest that the newly described direct association of paxillin with the integrin beta 3 tail is of physiological relevance.

3. Kindlin, talin, and paxillin are all found in nascent focal adhesions. Given the overlapping binding site of paxillin on integrin with kindlin, it is hard to imagine how paxillin would localize to integrin independent of kindlin. The only possibility to me is that paxillin and kindlin bind to different integrins on the cell surface while being localized to integrin site but there is not data in the manuscript to support this possibility.
Overall, while I think the in vitro binding analysis appears to be ok, the physiological relevance and the mechanism of such binding remain confusing.

As mentioned by the reviewer, it is indeed astonishing how the limited integrin beta tail can sustain such a high number of specific protein-protein interactions, which of course are not limited to talin, kindlin, and paxillin, but also have been reported for alpha-actinin, FAK, filamin, ICAP-1, DOK-1 and tensin, to name but a few (Legate, K.R., and R. Fässler. 2009. Mechanisms that regulate adaptor binding to beta-integrin cytoplasmic tails. J Cell Sci 122:187-198). While

some of these interactions are competitive, there are also examples of cooperative binding and recently, an unforeseen behaviour of asymmetric affinity regulation by integrin tail allostery has been described (Aretz J, Aziz M, Strohmeyer N, Sattler M, Fässler R. 2023. Talin and kindlin use integrin tail allostery and direct binding to activate integrins. Nat Struct Mol Biol. 30: 1913-1924. doi: 10.1038/s41594-023-01139-9). The biochemical data presented in that study suggest that kindlin binding to the integrin beta 1 tail increases the affinity of talin to associate with the same integrin tail, whereas the binding of talin in turn decreases kindlin's affinity for its neighbouring binding site on the integrin tail. This surprising finding suggests that while kindlin exhibits cooperative binding behaviour towards talin-binding to ITGB1, talin shows competitive behaviour towards kindlin-binding to ITGB1. However, despite the decreased kindlin-integrin affinity in the presence of talin, a ternary complex between integrin, talin and kindlin is observed, as this ternary complex is stabilized by direct talin-kindlin interactions. We also can envision that the flexible loop of paxillin LIM3 enables this protein to grab a minimal binding site encompassing the C-terminal amino acids of the integrin beta 3 tail, even though this binding site partially overlaps with the kindlin binding site and vice versa. However, such a presumably competitive behaviour with regard to direct integrin association might be compensated by the binding interactions between paxillin and kindlin/talin, which are mediated by paxillin's LIM4 and LD domains, respectively.

It has also to be mentioned that there are reports indicating that Focal Adhesion Kinase recruits talin to nascent adhesions (Lawson, C., S.T. Lim, S. Uryu, X.L. Chen, D.A. Calderwood, and D.D. Schlaepfer. 2012. FAK promotes recruitment of talin to nascent adhesions to control cell motility. J Cell Biol. 196:223-232) and in turn the focal adhesion localization of FAK depends on paxillin (Tachibana, K., T. Sato, N. D'Avirro, and C. Morimoto. 1995. Direct association of pp125FAK with paxillin, the focal adhesion-targeting mechanism of pp125FAK. J Exp Med 182:1089-1099). Accordingly, there could be different scenarios (depending on the integrin heterodimer and the cellular and extracellular matrix context), in which temporal order the core focal adhesion proteins are associating with the integrin beta tail. Accordingly and in reminiscence of the data reported by Aretz et al (Aretz J, Aziz M, Strohmeyer N, Sattler M, Fässler R. 2023. Talin and kindlin use integrin tail allostery and direct binding to activate integrins. Nat Struct Mol Biol. 30: 1913-1924. doi: 10.1038/s41594-023-01139-9), there could be an initial direct interaction of paxillin with the distal integrin beta tail, which could facilitate subsequent binding of kindlin and talin. At the same time, kindlin binding to the integrin tail could compete with the direct binding of paxillin and facilitate talin binding as reported by Aretz et al. (discussed above). Since paxillin could remain associated (due to its multipronged indirect associations) with the beta tail, one could envision even a quadruple complex of these core adhesome proteins. However, the molecular details of such putative complexes need to be explored in future studies.

In conclusion, the multitude of known biochemical interactions between integrins and core focal adhesion proteins together with our newly reported direct binding interaction between paxillin LIM2/LIM3 domains and the distal integrin beta 3 tail are not mutually exclusive, but rather provide our cells with a toolbox of matching scaffolds and adaptors to arrange and modulate the linkage between integrins and the cytoskeleton.

Reviewer #2: The paper by Baade et al. Report on the study of interactions of the paxillin LIM3 domain with an integrin protein. The paper contains cell-biological and biophysical experiments to probe for the interaction. The paper is well written, interesting to read and the conclusions are backed by data. The necessary controls have been mostly made. I consider this a careful study that should be published, and I have not detected any major issues.

I am not an expert on the cell-biological experiments but rather want to comment on the NMR part. I have a few minor suggestions:

* To better comprehend please color-code the linker in Fig. 2

We thank the reviewer for this suggestion. Accordingly, the linker region between LIM2 and LIM3, residues F438-A439-P440-K441, has now been colored in green in Figure 2B and 2C.

* More importantly, please show domain borders in the heteronuclear NOE data in Fig S2A. I assume that the linker is around residue 452 (?). However, I noticed 3 more regions of slightly increased flexibility, which is likely due to loops (around residues 392, 418 and 480). Only the latter is discussed in the text.

* How many interdomain contacts are observed as NOEs?

Thanks for all these suggestions! The domain borders/linker regions in the LIM2/3 domains, the alignment of the two LIM domains, and interdomain contacts have now been incorporated in the revised Suppl. Fig. S2 and/or in the text of the results section. Specifically, we discuss these structural features on page 10, line 180ff:

"*Not surprisingly regarding their high sequence similarity (39% identity when aligning residues P381-D436 of LIM2 with P440-E495 of LIM3; Suppl. Fig. S2B), both the LIM2 and the LIM3 domain of paxillin exhibit the characteristic double zinc finger motif as described for other LIM domain containing proteins and paxillin family members (Kadrmas and Beckerle, 2004; Kontaxis et al., 1998; Matthews et al., 2009; Perez-Alvarado et al., 1994)(Fig. 2B and C). Each domain comprises two orthogonally packed β-hairpins, followed by an α-helix (Fig. 2B and C). Interestingly, the LIM2 and LIM3 domains are connected by a short linker of 4 amino acids (F438-K441). There are in total only 19 long-range NOEs connecting two residues of the LIM2 domain (K432 and F435) with four residues of the linker region and the LIM3 domain, respectively (K440, R446, T458, and L459).*"

Regarding the flexible regions, the updated manuscript now reads on page 11, line 198ff:
"The heteronuclear NOE discloses further regions of increased flexibility in both domains, namely around K393, and E419 in the LIM2 domain, and E451, and S479 in LIM3. All these regions are located in loops leading from the first two ligands in each zinc finger to the following β-hairpins in each LIM domain, respectively."

*	Is the flexible loop in the LIM3 domain rigidified upon interaction with the integrin? Any change in the magnitude of the heteronuclear NOE for loop residues?

Regarding changes in dynamics of the flexible loop of LIM3, upon binding to integrin β3, we have recorded HetNOE spectra of the complex and re-recorded the spectra of free paxillin-LIM2/3. Unfortunately, with the samples and spectrometer equipment available, the differences between samples are within the error margins such that we cannot clearly identify a rigidification of the flexible loop of the LIM3 domain upon binding.

*	What is the sequence homology of LIM2 and LIM3? Do they stem from gene duplications?

The alignment of the two LIM domains has now been incorporated in the revised Suppl. Fig. S2B. Furthermore, the text and reference in the results section on page 17, line 360ff now reads:

“*The LIM2 and LIM3 domains share significant sequence homology with 39% identity and 53% similarity within 56 aligned residues. Regarding this high similarity and the tandem arrangement, the LIM domains of Paxillin most likely originate from gene duplication events. This has also been proposed by others on a broader context of LIM domain-containing proteins. (Winkelman et al., 2020)*”

*	Concerning the interaction studies: I realized that CSPs were much larger on the LIM domains than on ITGB3 (compare figures 2C and 3A). Please comment. Or does the interface involve aromatic residues on one side that would result in larger CSPs?

Thanks for pointing this out as this may cause confusion.
When comparing CSPs brought about by comparable concentrations of the non-labeled partner, as shown in the bar diagrams Fig. 2E (former Fig. 2D) and Fig. 3B, the CSP are overall rather similar in size (up to ca. 0.07 ppm @ ca. 750µM non-labeled interaction partner).
In the spectra shown in Fig. 3A, the final concentration of integrin-β3 was almost 3-fold higher than in the opposite case, which is depicted in Fig. 2D (2420 µM ITGB3 in Fig. 3A vs. 900 µM Pxn-LIM2/3 in Fig. 2D). Accordingly, one reason for the different size of the CSPs is the difference in concentration of the unlabeled interaction partner.
It may also play a role that the flexible loop of LIM3 is flanked by three phenylalanine residues (F475, F480 and F481) with F466 and Y493 being in the vicinity. Binding of the integrin peptide will change the conformation of the loop and slightly shift the arrangement of the phenylalanine sidechains. This, in turn, may lead to somewhat larger CSPs of residues within the loop.
It should also be noted, that some residues are, apparently, quite sensitive to slight changes in buffer composition as can be seen in Fig. 3C and D (e.g. F421, Y453 and F480). In the NMR-Titration of the Pxn-LIM2/3-4A variant, these residues showed large CSP. However, in this

case, these CSPs did not saturate and therefore do not indicate specific binding of the 4A-variant towards ITGB3.

To make these differences in the experimental conditions between Fig. 2C and Fig. 3A more obvious, we have modified the results section on page 13, line 251 of the revised manuscript to now read:

"It should be noted here, that the integrin β3 peptide can be added to higher final concentrations compared to the titration of 15N-labeled integrin β3 with unlabelled paxillin. Therefore, the maximum CSPs observed in Fig. 3A are larger than those in Fig. 2C. Changes in the arrangement of aromatic sidechains with respect to the amide groups of the flexible loop may play an additional role."

And later:

"The relatively large CSPs of a few isolated residues (F421, Y453 and F480) did not saturate and, therefore, do not indicate specific binding of the 4A-variant towards ITGB3. Instead, these residues seem to be quite sensitive to slight changes in buffer composition."

* Sometimes figures are differently in size. For example 3B and 3D show the same region on both axis but the figures are differently scaled so that a wrong impression about the relative CSPs is given, please make the plots identical in size.

Thanks for pointing this out. We apologize for this altered scaling of the panels, which seems to have occurred during the assembly of the Figure. The bar plots in Fig. 3B and 3D are now shown in exactly the same format.

* I always prefer to have an HSQC spectrum in the main paper because it tells a lot how well-behaved the protein is (fonts seem to be stretched in the horizontal direction in S2C).

Suppl. Fig. S2C has now been incorporated in Main Fig. 2 as the first panel (Fig. 2A)

* Which residues were used for the superposition for the calc of the bb RMSD in Table 1?

Thanks for pointing us to this omission! For LIM2, residues P381-F438, and for LIM3, residues P440-R497 have been used for superposition and calculation of the r.m.s.d. Table 1 has now been updated accordingly.

* Did the structure calculation comprise a refinement in explicit water? Which parameter were used as distances for the Zn coordination?

The 3D structure was calculated with CYANA without further refinement in explicit water. For Zn-coordination, distances and dihedral angles were constrained to values determined in high-resolution X-ray structures of Zink-binding proteins according to Alberts et al. (1998). This information, including the reference, has been added to the methods section

* Line 601: The authors name is "Güntert"

Thanks! This has now been corrected!

* Please insert a reference for the method to establish the coordination mode of the His residues

Thanks for pointing us to this omission!
We used the difference of the ^{13}C chemical shifts of $C^{\delta 2}$ and $C^{\varepsilon 1}$ to identify the coordination mode according to Barraud et al. (2012): Barraud P, Schubert M, Allain FH. (2012) A strong 13C chemical shift signature provides the coordination mode of histidines in zinc-binding proteins. J Biomol NMR. 53(2):93-101. doi: 10.1007/s10858-012-9625-6)
This information, including the reference, has been added to the Methods section

* Please add assignment statistics to Table 1 or elsewhere. Were all backbone atoms assigned?

Table 1 has been revised and all the information has been added. The backbone assignment was nearly complete (99%). Two amides (N477 and N505) were invisible probably due to exchange broadening. The extent of the resonance assignment over all residues was 90%.

* I am not an expert on this, but to which extent is a interaction of 530 uM biological significant (lines 203/204). Please comment.

This point has already been touched upon in the discussion section. Indeed, we are convinced that these relatively weak affinities are biologically relevant and the phenotypic effects in the cellular context also support such a view. We have expanded our discussion on page 18, line 380 as follows:

“The affinity of an individual interaction between paxillin and integrin β3 with KD ~ 500 µM is surprisingly weak. However, during FA assembly many of such interactions are formed, and during disassembly, all these contacts have to dissociate. Similar to many other well known, multivalent interactions, such as those mediated by oligosaccharide-lectin interactions in cell-cell recognition, such interactions may become – through avidity – very strong but still allow for fast assembly and disassembly through Velcro-like mechanisms.”

* The equation on line 664 has a typo. The "2" after the round brackets under the root is a square, not a factor of 2.

Thanks for the diligent checking! This has now been corrected.

Reviewer #3: Paxillin is one of the most important but also still highly enigmatic proteins, localizing to cell-matrix adhesions (also called focal adhesions), thereby controlling the critical mechanisms of cell spreading. Paxillin is composed to of a highly flexible N-terminal domain exhibiting several small structural-defined helical motifs with characteristic acidic and hydrophobic amino acids (LD-motifs). These LD-motifs can interact with other focal adhesion proteins such as talin, vinculin and focal adhesion kinase. Interestingly, these LD-motif interactions are considered of low-affinity, since the expression of the N-terminal domain alone, is not enabling the efficient recruitment of paxillin to integrin-containing focal adhesions. In addition the N-terminal domain contains a proline stretch forming a binding site for the src SH3 domain, potentially relevant for regulation of paxillin recruitment to focal adhesions by tyr-phosphorylation. In contrast to the highly flexible N-terminal domain, the focal adhesion-targeting domain of paxillin is localized in the c-terminal domain of paxillin, which is composed of 4 tightly spaced LIM-domains. Initial LIM-domain deletion studies by Brown and Turner (1996) have shown that deletions of LIM3 and to a minor level LIM2, cause a strong reduction in focal adhesion localization when transfected in fibroblasts. However, more recently, quantitative approaches to measure the individual contribution of the LIM-domains to beta3-integrin-containing focal adhesions, revealed a surprisingly non-sequence specific role of LIM3 in the paxillin recruitment mechanisms. The exchange of LIM3 with either LIM1 or LIM2, barely modified paxillin affinity to focal adhesions, while its deletion strongly affected it (Ripamonti et al., 2021). Instead of an adhesion targeting role in LIM3, a lipid-binding motif was detected in LIM4 and the strongest interference with focal adhesion recruitment was observed by exchanging the LIM2 and LIM1, with LIM3 domains.
This very old and the more recent studies set the stage for the current manuscript, but highlights also potential problems in the interpretation of the biological results, when focusing exclusively on the role of the LIM3 domain for paxillin recruitment to focal adhesions. Due to the central position of the LIM3-domain in the 4 LIM-domain array, slight perturbation of the LIM3 domain will cause paxillin recruitment defect that cannot be easily distinguished from a perturbation with a flexible integrin peptide. While direct integrin/paxillin LIM-domain interactions were never shown before, the interactions of NPXY-motif containing peptides with the LIM-domains from Enigma, and from 4-1/2LIM-domain proteins were reported. Based on these previous observations, we would expect the NPXY-motifs of integrins to bind to paxillin LIM domains, potentially binding to hydrophobic pockets present on the LIM-domains.

We thank the reviewer for this very detailed, expert description of the current state of research on paxillin's localization at focal adhesions. Prompted by this summary, we have tried to rephrase our introduction and have clarified several passages. The respective paragraph on page 7, line 114, now reads:

"*Paxillin is commonly employed as a marker protein of FAs and nascent focal complexes under various conditions, even where the normal morphology, function and architecture of FAs is disturbed. Paxillin is one of the first proteins recruited to FAs (Digman et al., 2008) and efficiently localizes there even in the absence of myosin-generated forces (Pasapera et al., 2010). Paxillin is found in nanometer distance from the plasma membrane with the carboxy-*

terminus detected in the same confined membrane-proximal layer as the cytoplasmic domain of integrin αv (Kanchanawong et al., 2010). A main determinant of paxillin's efficient recruitment to integrins seems to be its association with kindlin2, which has been mapped to the amino-terminal LD domains and the carboxy-terminal LIM3 / LIM4 domain region of paxillin (Bottcher et al., 2017; Gao et al., 2017; Zhu et al., 2019). Recently, a crucial role of the paxillin LIM2 domain for focal adhesion recruitment has been suggested (Ripamonti et al., 2019), while initial work identified the paxillin LIM3 domain as being the essential determinant of its focal adhesion targeting (Brown et al., 1996). A further mode of indirect recruitment of paxillin to integrin beta tails can be mediated by the interaction of paxillin LD motifs with the talin R8 rod domain (Zacharchenko T et al., 2016). However, paxillin can localize to FAs in talin knockout cells (Atherton et al., 2020) and paxillin is clearly incorporated into FAs in the absence of kindlins (Klapproth et al., 2019; Theodosiou et al., 2016) suggesting additional currently uncharacterized, kindlin- and/or talin-independent mode(s) of paxillin's integrin engagement."

We also agree with the reviewer that the numerous studies in this field have to be interpreted with caution and require careful consideration of the experimental conditions used (such as Mn^{2+} stimulation, matrix composition, overexpression or large deletions in the investigated proteins, use of modified integrin β3 cytoplasmic tails, etc.). However, we would like to stress at this point that the classic study by Brown and Turner (which we also cite and heavily rely on in our interpretation) does not only employ gross domain truncations, as eluded to by the reviewer. Indeed, Brown and Turner also use precise point mutations in different LIM domains (e.g. cysteine replacement to disrupt the Zinc-finger motif in paxillin's LIM2 domain or in paxillin's LIM3 domain). In this case, only disruption of the Zinc-finger motif in the LIM3 domain, but not in the LIM2 domain, alters focal adhesion targeting of paxillin, clearly stressing that a functional LIM2 domain or a functional LIM domain at position 2 of the LIM domain array of paxillin is not needed for focal adhesion recruitment.

Here in this manuscript the authors use NMR to probe the interaction of a purified LIM2/LIM3 tandem construct with either the beta1 and beta3 integrin tails. As expected the interactions are of low affinity, but sufficiently robust to evaluate the direct interaction of integrins with the LIM-domain of paxillin (surprisingly beta1 binds 10x better than beta3). By mutagenesis the authors identify a conserved and slightly flexible loop in LIM3 that is apparently relevant for binding to the very c-terminal end of the integrin b3-tail. They tested c-terminal deletions of 3 and 8 residues, in pull-down and cell-based assays with kindlin-deficient cells, as well as Ala-mutation of the loop residues in LIM3. When probing with N15-labeled integrin tails, perturbations by the Paxilin LIM2/LIM3 construct were found in the first and second NPXY-motif and at conserved inter-NPXY-residues for the beta3-tail, while for beta1 integrin tail, perturbations were identified only in the first NPXY-motif and inter-NPXY-residues, but not in the second NPXY-motif. Further c-terminal deletion of three residues in beta3, reduced paxillin pull-down, and completely blocked it when the 8 C-terminal residues were removed. Similarly paxillin 4A-substitution in the LIM3-loop only partially reduced beta3 binding, showing an alternative beta3 binding site in LIM2. Expression of beta3 integrins in kindlin ko cells partially rescued cell

spreading, by developing small and instable cell projections. These projections were lost when the 3 and 8 c-terminal residues were removed, showing a more severe cellular phenotype compared to the pull-down and NMR-perturbation experiment, showing partial interactions with the del3-construct. Beta3 integrin-deletion studies indicated a signaling function for the last three amino acids in beta-3 integrins, which is consistent with the previously identified c-src binding site (however not mentioned in the manuscript).
Based on these data the authors propose a conserved binding site for beta3-integrins in the LIM3 domain of paxillin, recognizing the C-terminal end of the integrin peptide. However, the direct link between paxillin and beta3 integrin binding at LIM3 is not that evident and the effect of c-terminal integrin deletions are not consistent with the effects of the pull-down assays. Instead, the data supports a critical signaling function of the c-terminal RGT sequence in beta-3, while showing only minor perturbation of paxillin pull-down. Similarly mutation of LIM3 leads to NMR perturbation in LIM2, while maintaining the integrin pull-down. Therefore the presented data are consistent with deletion of the src binding site in the c-terminal integrin tail, and a redundant or multiple LIM-domain spanning binding sites for integrin tails.

We appreciate the very careful review of our manuscript by Referee 3. However, in this case, the presentation of our data was apparently misleading and we hope that we can better explain our data in the revised version. We want to point out that we do see some CSPs within the LIM2 domain upon ITGB3 binding in the context of an intact LIM3 domain. However, in the Paxillin-LIM2/3-4A-variant, we do not see saturatable binding to either of the LIM2 or LIM3 domain. The CSPs visible in Fig. 3C and D (residues F421, Y453 and F480) therefore must not be interpreted as specific binding to LIM2 or LIM3. These effects rather reflect the sensitivity of some residues to slight changes of the buffer composition during the NMR titration. Therefore, our NMR data indicate a complete loss of integrin beta3 binding, when the LIM3-loop of paxillin is modified (paxillin 4A-substitution). We are now pointing this out more clearly in the revised manuscript on page 13, line 249:

“The relatively large CSPs of a few isolated residues (F421, Y453 and F480) did not saturate and, therefore, do not indicate specific binding of the 4A-variant towards ITGB3. Instead, these residues seem to be quite sensitive to slight changes in buffer composition.”

In summary, the quality of presented data is very good, but in multiple positions, the drawn conclusions are not supported by the data. This requires a considerable rephrasing of the text, a better and more balanced introduction to the field and a more careful interpretation of the data.

Major points:

Introduction:
While the general introduction into focal adhesions and the critical role of talin and kindlin is well done, it is important to mention that information gathered from studies in talin or kindlin-ko-cells are limited by their non-physiological context. Especially when Mn-dependent cell-matrix adhesions are introduced, the readers should be aware of the experimental nature of these

adhesions, failing to represent the regulatory complexity of focal adhesions. For example, when citing Atherton et al., 2020, where the known link between the paxillin N-terminus and vinculin tail, or talin-rod domains has been experimentally confirmed, the readers should be made aware that in talin ko cells, paxillin and vinculin recruitment to Mn-activated integrins was independent from each other, which is different from what is stated in the text (line 119). In lines 123 -125, the idea that kindlin directs paxillin to focal adhesion is well introduced, but the alternative vision that the proximal NPXY-motif in integrins is critical for paxillin recruitment and induction of cell spreading is not introduced (Pinon et al., cited in the discussion). In addition, the recent literature analyzing the differential roles for LIM2 and LIM3 for recruitment of paxillin to focal adhesions should also be introduce (Ripamonti et al 2021; cited in the discussion). In this work, a critical and sequence specific role for LIM2, but not for LIM3 has been demonstrated. This information is particularly relevant, as the authors show partial reductions in integrin pull-downs with the 4A-mutant of the LIM2/LIM3 tandem construct, which proposes additional LIM-domain-dependent interactions outside of the LIM3 domain (e.g. Fig 3C-E).

As mentioned above, we have rephrased the Introduction section to clarify the statements with regard to talin-deficient cells and also included the recent work by Ripamonti et al.
It should be noted here, that the pull-down experiments are performed with a construct that presents ITGB3 in a multivalent fashion. The decreased but not completely abrogated pull-down of Pxn-LIM2/3-4A is therefore not necessarily due to interactions with the LIM2 domain but could also be brought about by some residual affinity of the mutated LIM3 domain in the context of a multivalent, surface-associated binding partner.

Results:
Fig1. This new bacteria-based integrin clustering experiments nicely shows the integrin specificity of the paxillin family of LIM-domain containing proteins. This is an important experiment for the community as integrin-dependent (this report) versus tension-dependent (Schiller et al) LIM-domain recruitment mechanisms can be distinguished.

We thank the reviewer for this encouraging comment.

Fig2. NMR-based titration experiments are a very powerful technique to analyze low-affinity interactions between peptides and rigid protein domains. A general problem with low-affinity interactions is however the very high concentration of protein required for the measurement. High protein concentrations can reveal non-physiological effects within the protein solution. I found it surprising that the NMR-perturbation signal in the different titration curves for beta-3 integrin tail in Fig2C (inserts), is not saturating. This is different for the analysis with the beta1-integrin tail, potentially reflected by the 10 fold different affinity constant. The addition of a sentence mentioning this non-saturating behavior would be important, as it points to multiple less-definable interaction sites.

Thanks for bringing up this point. The integrin peptides used in this study can be concentrated up to ~2mM. The NMR titrations in Fig. 2C were performed up to a concentration of the integrin peptide of ~1.2 mM. Considering dilution effects that are almost unavoidable during NMR titration experiments, this is about as high as one can go with these binding partners. Indeed, with a K_D of 500 µM, it would be necessary to titrate up to 5 mM peptide concentration to achieve ca. 90% fraction bound and approach saturation. Nevertheless, at 1.2 mM peptide concentration, we have exceeded the KD value by almost 2.5-fold, and we observe a clear curvature of the titration curves. Therefore, we consider these titrations and the KD value derived thereof as reliable. To clarify, we have added the following sentence to the amended manuscript:

"As expected regarding the higher KD value in case of integrin β3, the titration curves do not reach full saturation but end at ca. 70% fraction bound. This is limited by the maximum concentration of unlabelled binding partner that could be reached and limits, in turn, the accuracy of the KD value determined."

It should be emphasized that our titration data is compatible with a one site binding model with moderate affinity. Our data do not indicate the presence of multiple / additional binding sites. Rather, we cannot exclude the presence of additional binding sites with lower affinity as this would require binding studies at higher peptide concentrations.
In our experiments with the Pxn-LIM2/3-4A-variant, we observed non-saturable behavior, i.e. a linear rather than curved relationship between CSP and peptide concentration up to c(Peptide) = 1.2 mM. If there were additional interactions their K_D values can therefore be expected to be above 1.5-2.0 mM, at least.

Fig2. The readers should be made aware of the discrepancy of the data shown in figure 2. While the NMR-perturbation experiments show a complete loss of interaction in the absence of the RGT-sequence in the beta3-integrin, there is still a considerable amount of LIM2/LIM3 pulled-down with the RGT-deleted integrin beta-3 peptide. Apparently, beta-3 integrin-peptide interactions at the first and second NPXY, as well as inter-NPXY-region are critically dependent on the RGT-peptide. Since Arg-residues are often involved in protein-protein or protein-peptide interactions, the specific mutation of the Arg-residue would have been a logic addition, especially since in beta1, a Glu-residue is following the second NPXY-motif, without inducing a relevant integrin/paxillin interaction.

We agree and indeed hope that our study will inspire / trigger many follow-up investigations including experiments with further mutated variants of the integrin cytoplasmic tails and paxillin's LIM domains. It is indeed intriguing that in case of integrin b1, the binding site is "shifted" to the region C-terminal of the first NPXY motif, and in future studies, the determinants of binding should be further specified.
On the other hand, there are positively and negatively charged residues among the 20 C-terminal amino acids of integrin β1 (2x E, 3x K) as well as integrin β3 (1x E, 1x K, 1x R), and the underlying recognition motif is certainly not obvious.

We do not see a discrepancy in the data shown in Fig. 2. NMR titrations and pull-down assays complement each other but are not the same thing. A simple explanation of the pull-down result would be that deltaRGT has still enough residual affinity in the multivalent context of the pull-down.

Fig.3. The NMR perturbation experiments with the N15-labeled LIM2/LIM3 tandem domain are very instructive. However, there is a circular argument hidden in the analysis, that should be better explained to the reader. While showing that point mutations in critical Phe-residues lead to entire LIM3-domain perturbation (see figure S4A and S4B; for F475A and F480A-mutant), the same F480-residue is flexible and its side chain can be found unusually solvent exposed when interacting with the integrin beta-3 peptide (Fig. 3B). Could this actually mean that the beta-3 peptide is denaturing the LIM3 domain in this loop, leading to extensive remodeling of the LIM3 domain structure. Unfortunately, the subsequently used 4A-mutation, is also affecting the NMR-signal of neighboring LIM3-residues quite broadly, very similar to the F480A mutation (Fig. S4F). Therefore, I am particularly concerned with the mutation of the Gly-residues in the 4A-mutant. Since folding of LIM-domains does not only require hydrophobic residues, but also requires tight turns formed by critically positioned gly-residues, it is well possible that the 4A-mutation is locally perturbing the folding of the LIM3 domain. Blocking the gly-residue with the 4A-mutation could have strong negative effects on the folding of the neighboring residues and affect the entre LIM3 structure in complicated ways.
Moreover, figures 3C,D show that in the presence of the 4A-mutation in LIM3, there are new LIM2/beta3 interactions appearing, suggesting that the specificity of the detected beta3 interaction with LIM3 are rather low. This should be clearly stated in the manuscript, to avoid confusion and misinterpretation by the readers.
The considerable LIM2 involvement in beta3-binding is also shown in Fig. 3E, where the pull down with the 4A-mutant shows still important interactions not consistent with a unique critical role of LIM3.

Thanks for inspecting our results carefully and with the demanded scepticism!
According to the HetNOE data, F480 shows increased dynamics compared to the more rigid secondary structures of Pxn-LIM2/3. However, our structure calculations result in a well defined conformation of F480 with the side chain packing against a moderately hydrophobic stretch formed by I455, S456 and A457. Consequently, only one side of F480 is solvent exposed. Most likely, it is this packing interaction that critically stabilizes the fold of the LIM3 domain and that is lost when mutating F480, e.g. to alanine.
It should also be noted that the preceding residues of F480 are much less well defined in the structural ensemble. The most flexible residues of the loop are G478 and S479, according to their heteronuclear NOE values. It is plausible that their flexibility affects the amide of F480 much more than its side chain. Noteworthy, the following residue, F481, is as rigid as residues in regular secondary structure.
It is important to realize that we have detected CSPs on amide resonances of residues of the loop. These indicate changes of the local chemical environment, usually through involvement in

binding and (slight) conformational rearrangements induced thereby. However, we have not determined the 3D structure of the LIM2/3 integrin β3 complex. Therefore, we have no information on the details of the conformational changes induced upon binding the peptide.
We want to emphasize that our data does not indicate a remodelling of the LIM3 domain upon interacting with the integrin peptides. To the contrary, the CSPs induced by binding are rather small, and most of the resonances of LIM3 (and of course LIM2) are unaffected indicating that the overall fold of the LIM2/3 domains remains very stable.

Different scenarios arose when mutating the residues of the loop region, as described in the manuscript and shown in Suppl. Fig. S4A-F. Here, the interpretation of Referee 3 is not entirely correct. When mutating individually F475 or F480 or F481 to alanine, we observe very strong CSP all over the LIM3 domain with many resonances vanishing completely. This indicates complete or nearly complete unfolding of the LIM3 domain. In line with this, these mutants were hard to prepare, and were obtained in rather low yield, most likely because of protein aggregation and/or proteolysis.
Noteworthy, the resonances of the LIM2 domain remained largely unaffected by these dramatic changes within its neighbouring domain, which would argue that these two domains are structurally uncoupled.

When mutating the other residues of the loop and similarly in case of the 4A-mutant, there are also CSPs with some of them going up to 0.4 ppm, ca. 7-fold stronger than what is caused by integrin peptide binding. However, these are largely limited to the direct vicinity of the loop. In case of the 4A-mutant, sizable CSPs (up to 0.4 ppm) are observed within a stretch of at max 20 residues from 465 to 485, and almost no resonances vanish. In addition, this construct expresses in good yield and is stable in solution at similarly high concentrations as the wild type. For comparison, in F475A, F480A, and F481A strong CSPs start at the domain border and/or many resonances of the LIM3 domain vanish completely.

We have no indication from CSPs that the 4A-mutant undergoes structural remodelling. The replaced glycine is not located in a tight turn. Nevertheless, it would have been interesting to explore other replacements instead of the standard alanine mutation and this will surely be one of the next directions to be explored.

Regarding the additional / new interaction sites and the pull-down results mentioned by Referee 3, we refer to our comments from above.

In conclusion, we do not see a circular argument, here!

Fig. 4. Two different knockouts strategies are used in this figure to show the importance of the integrin c-terminal domain and that of kindlin, known to bind to this domain. Unfortunately, this figure is disconnected from the rest of the paper and an obvious link to the previously analyzed paxillin/integrin interaction cannot be drawn. Deletion of the RGT-sequence in beta3-integrin reduces spreading, while completely blocking it in the 8 aa-deletion. Although not specifically

analyzed, the deletion of RGT, may affect kindlin-recruitment, and kindlin-mediated cell spreading. Therefore causing a spreading defect because of reduced kindlin-binding rather than paxillin recruitment. This dependence on kindlin for cell spreading is confirmed by the removal of kindlin, blocking spreading of both the RGT- and 8aa- integrin deletion constructs. Thus kindlin is more relevant than a potential paxillin binding at the c-terminal integrin sequence. Previous work by Theodosiou et al., 2016, has shown that in the absence of kindlin2, paxillin is no longer recruited to Mn-induced talin-containing adhesions (please also correct the section in the introduction, where it is incorrectly stated that paxillin is recruited to talin, in kindlin-ko cells, the reverse has been observed by Theodosiou). According to the pull-down in the previous figures, paxillin should still be recruited to kindlin-deficient beta-3 wt or delta-RGT-integrin expressing cells, which is not the case from the images shown in Fig. 4F (while talin accumulation can be seen in the projections in figure 4F (right panel). Thus the biochemical data is not directly translatable to the observations made in kindlin-ko cells, and this should be clearly said in the manuscript, in order to avoid overinterpretation of the data.

We understand the reasoning of the reviewer and agree that it is not easy to disentangle the direct interaction of paxillin (binding site in the C-terminal region of ITGB3) from the known indirect interaction of paxillin with the integrin C-terminus mediated via kindlin-1 and kindlin-2. It is exactly for this reason that we combined two genetic manipulations in a single cell model: starting from genetically kindlin1/kindlin-2 deficient cells we have introduced either WT ITGB3 or the truncated (Δ3; Δ8) ITGB3 variants. Please keep in mind that the kindlin1/2 double knock-out cells exhibit strongly reduced levels of endogenous integrin beta3 (which was already reported by Theodosiou et al, 2016, and which we confirmed by flow cytometry). This low backgound of endogenous ITGB3 allowed us to re-express either the wildtype ITGB3 or its truncated versions and to test the role of the direct interaction of paxillin and ITGB3 in the absence of any kindlin-mediated indirect association.
Accordingly, we can attribute the observed gain-of-function by ITGB3-WT expressing cells with regard to cell spreading on the ITGB3 ligand to the direct interaction of paxillin with the far C-terminus of ITGB3, as this kindlin-independent spreading and paxillin recruitment is completely absent in ITGB3 Δ3 and ITGB3 Δ8-expressing cells. Therefore, we do not think that this experiment is disconnected from the rest of the paper, but it is a clear-cut demonstration that in addition to the known kindlin-dependent association of paxillin with the integrin cytoplasmic tail, a kindlin-independent recruitment of paxillin to ITGB3 is possible and we delineate the molecular details of this interaction in our study.
Our findings (and our statement in the introduction) are also completely in line with the report by Theodosiou et al. (Theodosiou et al. eLife 2015;5:e10130. DOI: 10.7554/eLife.10130) and we cite here from the results section of this paper:

“Interestingly, mature FAs in K2ΔPHGFP-expressing cells [expressing a kindlin mutant lacking paxillin binding] were prominent after 30 min and contained significant amounts of paxillin, **indicating that paxillin is recruited to mature FAs in a kindlin-2-independent manner** (Figure 4F).”

In this context, it is important to mention that Reviewer 1 brought up the point that under these circumstances (paxillin localization to FAs in kindlin1/2-deficient cells) there might still be a chance that the recruitment of paxillin to ITGB3-WT is mediated via an association of paxillin with talin. Reviewer 1 reasoned that potentially the integrin association of talin is affected by the C-terminal deletions of ITGB3 and that the failure of talin to associate with truncated ITGB3 variants could explain the lack of paxillin recruitment in these cells (see comment #1 of Reviewer 1).
To address this issue, we have now performed additional pull-down and NMR titration experiments with the talin FERM domain and integrin beta3 cytoplasmic tails, either in the WT form or in the form of the ITGB3 Δ3 / ITGB3 Δ8 variant. The novel data, which are now presented in Suppl. Fig. S3G and Suppl. Fig. S3H, clearly demonstrate that the talin F3 domain associates, as expected, with wildtype and truncated ITGB3 cytoplasmic domains. Therefore, the spreading of ITGB3-WT-expressing, kindlin2/3 deficient cells on vitronectin (and the lack of spreading by the corresponding ITGB3 Δ3 / Δ8-expressing cells) is best explained by the direct association of paxillin's LIM domains with the C-terminus of ITGB3

Minor points

In line 219, the beta3-S778A mutation is mentioned, without citing or showing the data that this mutation would induce a kindlin-binding defect. Likewise, potential kindlin-binding defects to the del RGT-sequence are not shown, but are key for understanding the experiments in figure 4.

We have to apologize for this labeling mistake, as we have employed a ITGB3 S778P mutation, which we had wrongly labeled in Suppl. Fig. S3E and S3F. We have now corrected this mistake in the respective figure panels. The beta3-S778P mutant is identical to the S752P mutant investigated by Ma et al. (Ma YQ, Qin J, Wu C, Plow EF. Kindlin-2 (Mig-2): a co-activator of beta3 integrins. J Cell Biol. 2008 May 5;181(3):439-46. doi: 10.1083/jcb.200710196) and Moser et al. (Moser M, Nieswandt B, Ussar S, Pozgajova M, Fässler R. Kindlin-3 is essential for integrin activation and platelet aggregation. Nat Med. 2008 Mar;14(3):325-30. doi: 10.1038/nm1722) and shown to disrupt Kindlin3-binding to ITGB3. The same missense mutation is also found in some patients with Glanzmann's thrombasthenia (Chen, Y.P. et al. (1992) Ser-752-Pro mutation in the cytoplasmic domain of integrin b3 subunit and defective activation of platelet integrin aIIbb3 (glycoprotein IIb-IIIa) in a variant of Glanzmann thrombasthenia. Proc. Natl. Acad. Sci. USA 89, 10169–10173).
Our amino acid count is based on the ITGB3 protein sequence available on Uniprot (https://www.uniprot.org/uniprotkb/P05106/entry). Therefore, our amino acid count deviates from the one used by Moser et al. and Ma et al., as they do not include the 26-amino acid signal sequence in their amino acid numbering.
We now cite the papers by Ma et al. and Moser et al. at the appropriate position in the manuscript (page 12, line 239).

In line 244, the use of the paxillin delta-LIM4 construct is mentioned as control for a perturbed LIM4/kindlin interaction. Considering that the LIM4 deletion is mainly affecting membrane-interaction and has only modest effects on the recruitment of paxillin to focal adhesions, this control is not able to distinguish between a kindlin, or integrin-dependent paxillin recruitment mechanisms.

In this regard, we refer to the publication by Zhu et al. (Zhu, L., H. Liu, F. Lu, J. Yang, T.V. Byzova, and J. Qin. 2019. Structural Basis of Paxillin Recruitment by Kindlin-2 in Regulating Cell Adhesion. Structure. 27:1686-1697 e1685.), which report the structure and the functional connection between Kindlin2 and the paxillin LIM4 domain. These authors also show a diminished focal adhesion localization of paxillin mutants, which harbor point mutations in the LIM4 domain disrupting the binding to Kindlin F0-domain. Given that also other researchers have pinpointed the interaction of kindlin to the LIM4 domain of paxillin based on biochemical crosslinks (Bottcher, R.T., M. Veelders, P. Rombaut, J. Faix, M. Theodosiou, T.E. Stradal, K. Rottner, R. Zent, F. Herzog, and R. Fassler. 2017. Kindlin-2 recruits paxillin and Arp2/3 to promote membrane protrusions during initial cell spreading. J Cell Biol. 216:3785-3798) we believe it is fair to state that the deletion of the paxillin LIM4 domain can perturb the paxillin/kindlin interaction. We are aware that the LIM4 domain has also been reported to mediate membrane binding of paxillin and we have added this information.

When analyzing the flexibility of the LIM2/LIM3 construct by NMR, there is a similar flexible loop observable in LIM2 (Fig. S2). The authors should comment on this similarity, and discuss how solvent exposure of the flanking Phe-side chains as seen in the NMR structure, could perturb the overall folding of the LIM domains.

Thanks for bringing up this point!
Indeed, there are additional flexible regions and, in particular, a similarly flexible loop in the LIM2 domain. These regions are now described in the amended manuscript and the corresponding flexible loops in LIM2 and LIM3 have been color coded in Fig. 2B and C.
The flexibility of the loop in LIM2 is now described on page 11, line 211ff:

"*Noteworthy, the LIM2 domain harbors a similarly flexible loop, yet with a negatively charged residue at its center (E419) and a less hydrophobic patch opposing it (LIM2: K393VVTALD399 vs. LIM3: N452YISALN458, Suppl. Fig. S2B).*"

In addition, we have expanded the display of the sequence alignment in Fig. S2B to include a direct comparison of the LIM2 and LIM3 domains of human. Despite similarities, there are marked differences between LIM2 and LIM3 in their flexible loops (LIM2: F_{416}GPEGFH_{422} vs. LIM3: F_{475}VNGSFF_{481}) with the LIM2 domain exhibiting a negative charge and a conformation-restricting proline residue. Furthermore, one of the flanking phenylalanines (F416) has its side chain fully solvent exposed. Nevertheless, in case of the LIM2 domain, this does not seem to lead to instability. No doubt that mutational studies would be interesting in this regard.

In summary, the manuscript shows a series of very interesting observations that are helpful for analyzing the mechanisms of LIM-domain-dependent recruitment of paxillin to integrin-cytoplasmic tails. Unfortunately, this reviewer is under the impression that the fixation on the likely overstated role of LIM3 for paxillin recruitment has prevented the authors from reporting potentially relevant interactions with the LIM2-domain of paxillin. In addition to the kindlin-mediated recruitment of paxillin, the direct interaction with integrin peptides is still quite obscure (see also Pinon et al., 2014) and the specificity and role of a direct integrin interaction with paxillin needs to be analyzed together with a proper discussion of the literature analyzing integrin/paxillin dependent cell spreading. In this context, the observed differences between beta1 and beta3 integrins are very interesting and need to be further discussed, and linked to the reported association of src with the c-terminal beta3-integrin tail.
