# Supplementary material for: A flexible loop in the paxillin LIM3 domain mediates its direct binding to integrin β subunits
Source: PLoS Biol. 2024 Sep 4;22(9):e3002757. doi: 10.1371/journal.pbio.3002757 (PMC11374337; doi:10.1371/journal.pbio.3002757)
Supplement: Attachment — Submitted filename: Response_to_reviewer_comments-7-2024.pdf [file pbio.3002757.s010.pdf]

Reviewer remarks:

Reviewer 1:

My major concern on the manuscript was the physiological relevance of the observed paxillin/integrin beta tail interaction in this manuscript. Moreover, given the potential competition between kindlin and paxillin for binding to the same region of integrin beta tail, I was concerned about the authors' statement of "kindlin-independent" paxillin-integrin interaction in regulating integrin function. The authors have made significant effort in addressing my concerns and acknowledged that there is competition between kindlin and paxillin for binding to integrin but explained that there is possibility of cooperative binding for such competition and the authors used the recent paper of talin/kindlin binding to integrin as an example to support such possibility (Aretz et al., NSMB, 2023). Honestly, it is hard to imagine how such cooperation can occur to the same integrin given the competition between kindlin and paxillin without definitive biological data. Nevertheless, I agree the observed paxillin/integrin beta tail interaction is a novel phenomenon but my concern of its physiological relevance still remains. I am fine for the journal to publish this finding and hopefully it will trigger some follow-up biological studies to prove its physiological relevance. The authors should further revise the manuscript by paying attention to the following:

1. The arguments provided in the response letter to address the physiological relevance should be properly and concisely added into the discussion.

We thank the reviewer for this suggestion and have added the respective paragraph now on page 19, line 383 to read:

"On first sight, such a cooperative binding scenario seems implausible as the binding sites of paxillin and kindlin at the carboxy-terminus of the integrin β3 subunit partially overlap. However, a recent study has highlighted an unforeseen behaviour of asymmetric affinity regulation by integrin tail allostery, where kindlin binding increases the affinity of talin to associate with the same integrin tail, whereas the binding of talin in turn decreases kindlin's affinity for its neighbouring binding site (Aretz et al., 2023). This surprising finding suggests that while kindlin exhibits cooperative binding behaviour towards talin-association with integrin β1, talin shows competitive behaviour towards kindlin-binding to the same integrin tail. However, despite the decreased kindlin-integrin affinity in the presence of talin, a ternary complex between integrin, talin and kindlin is observed, as this ternary complex is stabilized by additional talin-kindlin interactions. We also can envision that the flexible loop of paxillin LIM3 enables this protein to associate with a minimal binding site encompassing the C-terminal amino acids of the integrin β3 tail, even though this binding site partially overlaps with the kindlin binding site and vice versa. However, such a presumably competitive behaviour with regard to direct integrin association might be compensated by the binding interactions between paxillin and kindlin/talin, which are mediated by paxillin's LIM4 and LD domains, respectively."

2. Paxillin not only binds to integrin beta tail as shown in this manuscript but also to integrin alpha tails as shown previously by Ginsberg group (Liu et al., 2001, 2002, JBC). This is important and should be cited and discussed as to how paxillin might engage both integrin alpha/beta tails to regulate integrin function given that integrin functions as obligate alpha/beta heterodimer. Functionally, does paxillin bind both integrin alpha and beta tails simultaneously to promote FA assembly?

The reviewer is completely correct in pointing to a potential further direct association of paxillin with integrin heterodimers by the interaction of the paxillin N-terminus with the integrin alpha subunit as reported by Ginsberg and colleagues (e.g. Liu S, Kiosses WB, Rose DM, Slepak M, Salgia R, Griffin JD, Turner CE, Schwartz MA, Ginsberg MH. A fragment of paxillin binds the alpha 4 integrin cytoplasmic domain (tail) and selectively inhibits alpha 4-mediated cell migration. J Biol Chem. 2002; 277(23):20887-94. doi: 10.1074/jbc.M110928200.). However, as demonstrated in their initial report, this direct association of paxillin is specific for the integrin alpha 4 subunit, a protein specifically expressed in leukocytes, and has not been observed for other alpha subunits such as the alpha 5 subunit (Liu, S., S.M. Thomas, D.G. Woodside, D.M. Rose, W.B. Kiosses, M. Pfaff, and M.H. Ginsberg. 1999. Binding of paxillin to alpha4 integrins modifies integrin-dependent biological responses. Nature. 402:676-681). As the alpha4 subunit is not present in the cellular systems employed in our study, we have not eluded to these findings.

Nevertheless, we think the comment by the reviewer points to a highly interesting direction for further studies, as the paxillin binding site in alpha4 seems to be responsible for some of the peculiar features of α4β1 integrin-mediated cell adhesion in leukocytes, such as stimulation of cell migration, while opposing cell spreading and focal adhesion formation. It is highly interesting to speculate that the simultaneous direct binding (and thereby clamping) of the alpha4 subunit (reported previously) and the beta1 subunit (reported here) by paxillin might interfere with the "regular" recruitment of kindlin (responsible for cell spreading and further focal adhesion formation), thereby producing the peculiar phenotype of α4β1 integrin-mediated cell adhesion.

3. The title is confusing. Why is direct LIM3 binding to integrin b3 only? But the short running title states paxillin binding to integrin beta subunits. Given that paxillin binds much tighter to integrin b1 tail, the title may be misleading to the readers that paxillin only mediates integrin b3 function.

We have followed the advice of the reviewer and the Journal editor and changed the title to: "*A flexible loop in the paxillin LIM3 domain mediates its direct binding to integrin β subunits*".

Reviewer #2:

all points I have raised in the previous review have been sufficiently been dealt with (in fact, they were answered very thoroughly)

We highly appreciate your expert insight and advice, which helped us to improve the manuscript.

Reviewer #3:

Suggested corrections to revision by Baade:

The revised version of the manuscript has clearly improved, as the introduction is more balanced and many of the experimental results better explained, or at least interpreted so that it fits the model of the authors.

However there is still a conceptual issue that is not completely resolved. Considering the fundamental nature of the paxillin family adapters and the strong sequence conservation in the LIM domains, it is surprising to note that the very similar cytoplasmic tails of beta1 and beta3 integrins show differences in the mode of paxillin recruitment. Since the identified RGT sequence in the beta3 is not conserved in beta1 integrins, it needs to be stated that the paxillin binding site found in this manuscript, may not reflect a typical paxillin recruitment mechanisms, but rather a specific feature of beta3 integrin.

Previously a beta3 integrin specific recruitment of the src kinase has been identified by Shattil and coworkers, involving the exact same RGT-motif in beta3 integrin. As this RGT-motif is critical for beta3-mediated recruitment of src for potentially inducing cell adhesion and spreading, it is possible that some of the observed cell spreading defects observed by the authors are due to a failure of beta3 to recruit src and not paxillin.

When analyzing the beta3-del3 mutation, the reduction in paxillin binding is partial, as is the recruitment of the paxillin LIM3-4A mutation. This translates into partial defects in spreading in beta3 and paxillin ko backgrounds. A more severe phenotype is observed upon deletion of the last 8 amino acids, which fits with the binding site of the kindlin adapter in the beta3 integrin, as well as the more extensive integrin beta3 binding site for paxillin.

In the absence of kindlin, the 3-amino acid deletion in beta3 is sufficient to completely perturb the beta3 induced cell adhesion and spreading. The enhanced severity of the phenotype in kindlin-ko cells shows the critical role of kindlin in the spreading process.

As the src binding site is much more confined to the RGT-sequence and not spanning 8-10 amino acids of the c-terminal end of the beta3 integrin, it is well possible that the failure of src binding to the deleted RGT sequence contributes to the strong spreading phenotype in the beta3 deletion mutants in the kindlin ko-background.

Currently information on the src-binding to the RGT-sequence are missing from the manuscript. As the below mentioned reports propose an alternative, paxillin independent explanation for the phenotypes of the beta3-deletion mutants they should be discussed. I would propose that the authors add a short paragraph (for example at line 425 in the discussion), and to mention that the last three amino acids specific to beta3 integrins "RGTcooh" have been shown to bind to the SH3 domain of Src,

providing a possible mechanism to provide an integrin beta3-specific ability to induce spreading in the absence of kindlin. (Xiao et al. Blood, 2013, 121, 700-706; Katyal et al. Protein Science, 2013, 22, 1358-1365).

We thank the reviewer for this important advice. While the C-terminal residues of the integrin β3 tail are clearly critical for the direct interaction with the paxillin LIM domains under *in vitro* conditions with purified, recombinant reagents, one should be careful with the interpretation of the *in vivo* consequences of expressing integrin β3 truncation mutants in a cellular context, such as the kindlin1/2 ko cells. Indeed, the lack of Src family PTK binding and activity could contribute to the observed phenotype and we now acknowledge this fact in the discussion section. We would like to state that the opposite is also true, as some of the phenotypes of integrin β3 truncation mutants attributed formerly to Src PTKs could, based on our data, also reflect a lack of direct association of paxillin with the integrin β3 tail. The relative contribution of these two binding partners under physiological conditions needs to be dissected in future investigations. Nevertheless, based on the suggestion of the reviewer, we have added the following sentences to the discussion section on page 22, line 443, as indicated by the reviewer:

"The C-terminal amino acids of integrin β3 differ from all other β subunits making this a unique recognition site and helping to explain this peculiar interaction mode of paxillin and integrin β3. These residues can also serve as an unconventional binding site for the SH3 domains of various Src family kinases, adding to Src kinase activation upon integrin engagement (Arias-Salgado et al., 2003; Katyal et al., 2013; Xiao et al., 2013). Accordingly, a reduction in Src kinase activity could also contribute to the spreading phenotype of kindlin knock-out cells expressing truncated integrin beta3 variants."

Minor issues :

Line 126: should read Ripamonti et al., 2021

corrected.

Line 271: The authors should indicate what changes in the buffer modified these residual peaks in the 4A-mutant (salt, and or pH?).

Of course, identically composed buffer solutions were used for preparing the protein and peptide samples. However, either due to differences during purification and or up-concentrating the samples, slight variations of pH and/or salt concentration may occur that affect the NMR titration results.

We have added this explanation now to the text on page 14, line 272:

“Though identical buffer solutions during NMR sample preparation were used, such differences may occur during up-concentration or as a consequence of variations within the purifications protocols of paxillin-LIM2/3 vs. the integrin peptides.”

Line 277: Please introduce the citation

The correct citation was added:

“In addition, we generated a truncated paxillin lacking the LIM4 domain (GFP-PXN ΔLIM4) to interfere with the kindlin-mediated indirect binding of paxillin to the integrin beta subunit as reported by Zhu et al. (Zhu et al., 2019).”
