# Supplementary material for: A flexible loop in the paxillin LIM3 domain mediates its direct binding to integrin β subunits
Source: PLoS Biol. 2024 Sep 4;22(9):e3002757. doi: 10.1371/journal.pbio.3002757 (PMC11374337; doi:10.1371/journal.pbio.3002757)
Supplement: Attachment — Submitted filename: Revision-1-review.docx [file pbio.3002757.s009.docx]

My major concern on the manuscript was the physiological relevance of the observed paxillin/integrin beta tail interaction in this manuscript. Moreover, given the potential competition between kindlin and paxillin for binding to the same region of integrin beta tail, I was concerned about the authors’ statement of “kindlin-independent” paxillin-integrin interaction in regulating integrin function. The authors have made significant effort in addressing my concerns and acknowledged that there is competition between kindlin and paxillin for binding to integrin but explained that there is possibility of cooperative binding for such competition and the authors used the recent paper of talin/kindlin binding to integrin as an example to support such possibility (Aretz et al., NSMB, 2023). Honestly, it is hard to imagine how such cooperation can occur to the same integrin given the competition between kindlin and paxillin without definitive biological data. Nevertheless, I agree the observed paxillin/integrin beta tail interaction is a novel phenomenon but my concern of its physiological relevance still remains. I am fine for the journal to publish this finding and hopefully it will trigger some follow-up biological studies to prove its physiological relevance. The authors should further revise the manuscript by paying attention to the following:

1. The arguments provided in the response letter to address the physiological relevance should be properly and concisely added into the discussion.
2. Paxillin not only binds to integrin beta tail as shown in this manuscript but also to integrin alpha tails as shown previously by Ginsberg group (Liu et al., 2001, 2002, JBC). This is important and should be cited and discussed as to how paxillin might engage both integrin alpha/beta tails to regulate integrin function given that integrin functions as obligate alpha/beta heterodimer. Functionally, does paxillin bind both integrin alpha and beta tails simultaneously to promote FA assembly?
3. The title is confusing. Why is direct LIM3 binding to integrin b3 only? But the short running title states paxillin binding to integrin beta subunits. Given that paxillin binds much tighter to integrin b1 tail, the title may be misleading to the readers that paxillin only mediates integrin b3 function.
